# Development and Validation of a Viability RT‐qPCR Assay for Detecting Infectious Spring Viraemia of Carp Virus (SVCV)

**DOI:** 10.1111/jfd.70163

**Published:** 2026-03-15

**Authors:** Ha‐Jeong Son, Min‐Young Sohn, Jae‐Ok Kim, Hee Jung Choi, Mun‐Gyeong Kwon, Jeong‐Tae Lee, Gyoungsik Kang, HyeongJin Roh, Chan‐Il Park, Kyung‐Ho Kim

**Affiliations:** ^1^ Department of Marine Biology and Aquaculture, College of Marine Sciences Gyeongsang National University Tongyeong Republic of Korea; ^2^ Aquatic Disease Control Division, National Fishery Products Quality Management Service Busan Republic of Korea; ^3^ Gyeongsangnam‐Do Fisheries Safety Technology Institute Tongyeong Republic of Korea; ^4^ Department of Aquatic Life Medicine, College of Marine Sciences Gyeongsang National University Tongyeong Republic of Korea

**Keywords:** aquatic virus, diagnostic performance, infectivity, quantitative PCR assay, viability PCR

## Abstract

Spring viremia of carp virus (SVCV) is a contagious pathogen associated with significant mortality and economic losses in freshwater aquaculture. Although reverse transcription quantitative PCR (RT‐qPCR) enables rapid detection, it does not distinguish between infectious and non‐infectious viral particles, which may lead to overestimation of infection risk. In this study, a viability RT‐qPCR (vqPCR) assay targeting the glycoprotein (G) gene of SVCV was developed and analytically evaluated. Primers and probes were designed based on 45 full‐length G‐gene sequences representing genogroups Ia–Id to ensure broad genetic coverage. The assay achieved a 95% limit of detection (LoD_95%_) of 6.82 copies per reaction and showed no cross‐reactivity with other tested aquatic pathogens. Optimisation using 25 μM PMAxx effectively reduced amplification from inactivated virus and free viral RNA while preserving detection of infectious SVCV. Under controlled laboratory conditions, the vqPCR assay successfully differentiated varying proportions of infectious virus in mixed samples. In laboratory‐simulated environmental freshwater, both qPCR and vqPCR signals declined with increasing temperature; notably, vqPCR reflected the loss of infectivity earlier than qPCR. The assay demonstrated good repeatability and reproducibility across three independent laboratories and showed overall agreement with cell culture and WOAH‐recommended nested PCR results. Although further validation under diverse environmental conditions is warranted, these findings indicate that the proposed vqPCR approach may serve as a complementary method for monitoring infectious SVCV in freshwater aquaculture.

## 1 Introduction

Spring viremia of carp virus (SVCV) is a negative‐sense RNA rhabdovirus with an −11‐kb genome that encodes five structural proteins (N, P, M, G and L) (Chen et al. 2008). Based on glycoprotein (G) gene sequences, SVCV comprises four genogroups (I–IV), with genogroup Ia predominating in Asia and the United States and being associated with outbreaks in the Republic of Korea (Stone et al. 2003; Padhi and Verghese 2012; Ashraf et al. 2016). Since its first isolation in Europe in the 1970s (Fijan 1973), SVCV has been reported across Europe, Asia and North America and can cause up to −90% mortality in susceptible cyprinids, particularly juveniles at spring temperatures of 10°C–17°C (Ahne et al. 2002; World Organisation for Animal Health (WOAH) 2023). Owing to its high transmissibility and economic impact, spring viremia of carp (SVC) is notifiable to the World Organisation for Animal Health (WOAH).

Diagnostic approaches for SVC include conventional PCR, sequencing, immunohistochemistry, antigen‐based ELISA, indirect fluorescent antibody test (IFAT) and virus isolation in cell culture. WOAH recommends RT‐PCR as the primary screening tool, with confirmation by inoculation into susceptible cell lines (e.g., EPC, FHM, GCO) followed by cytopathic‐effect (CPE) observation and molecular analyses (World Organisation for Animal Health (WOAH) 2023). However, PCR may amplify nucleic acids from non‐infectious or inactivated particles, potentially overestimating the presence of infectious particles (Rahman et al. 2013; Braunstein et al. 2021). Although cell culture remains a reliable method, variability in cell‐line susceptibility, long turnaround times, contamination risk and facility requirements limit its practicality for rapid diagnostics.

Viability RT‐qPCR (vqPCR) assay has emerged to address these limitations. This approach employs intercalating dyes such as propidium monoazide (PMA) or ethidium monoazide (EMA), which permeate only membrane‐ or capsid‐compromised (non‐viable/non‐infectious) targets and, upon photoactivation, irreversibly bind to nucleic acids, thereby preventing amplification from non‐viable targets (Nocker and Camper 2009; Fittipaldi et al. 2012). Recently, vqPCR assay has been applied in aquatic virology; for example, to selectively detect infectious red sea bream iridovirus (RSIV) and white spot syndrome virus (WSSV), highlighting its potential to differentiate viable from non‐viable viral particles in aquatic hosts (Kim et al. 2023; Shin et al. 2025).

In this study, we first developed and analytically validated a qPCR assay for the detection of SVCV, establishing its sensitivity and specificity as a molecular diagnostic tool. Building on this foundation, we optimised PMA treatment parameters including dye concentration and photoactivation time to adapt the assay into a vqPCR assay. The specificity and selectivity of the vqPCR assay were subsequently evaluated using culture‐confirmed infectious samples alongside non‐infectious samples inactivated by sodium dodecyl sulfate (SDS) and heat treatment, which selectively disrupted the viral envelope without completely degrading the viral genome. Furthermore, we compared the performance of the vqPCR assay with that of qPCR to assess its accuracy in detecting infectious particles. Lastly, the applicability of the assay to freshwater samples was assessed under laboratory‐simulated conditions. These findings offer preliminary insight into the use of vqPCR as a complementary method for detecting infectious SVCV, indicating a potential, albeit laboratory‐limited, utility for water‐based viral detection.

## 2 Materials and Methods

### 2.1 Development of an SVCV qPCR Assay

#### 2.1.1 SVCV‐Specific Primer and Probe Design

Specific primers and a hydrolysis‐probe set for SVCV were designed based on the glycoprotein (G) gene (1530 bp) sequences retrieved from the National Center for Biotechnology Information (NCBI) GenBank database. The dataset comprised 45 complete G gene sequences from diverse SVCV isolates: 29 of genotype Ia, 1 of genotype Ib, 7 of genotype Ic and 8 of genotype Id. Primer and probe sets suitable for qPCR assays were designed using the Primer3Plus software (Untergasser et al. 2012; Figure S1). To verify target specificity, in silico cross‐reactivity analyses were performed using the UCSC Genome Browser (https://genome.ucsc.edu/) and Primer‐BLAST (https://www.ncbi.nlm.nih.gov/tools/primer‐blast/) (Ye et al. 2012). The Simulate PCR tool (http://sourceforge.net/projects/simulatepcr) was employed to assess expected target amplification and potential non‐target cross‐amplification using SVCV sequences from the GenBank database (Gardner and Slezak 2014). In addition, sequence alignment of G‐gene sequences obtained from GenBank was conducted using Jalview v2.11.2.2 (Waterhouse et al. 2009) to confirm primer and probe identity and specificity for SVCV detection. qPCR assays were conducted using the Thermal Cycler Dice Real‐Time System III (Takara, Japan). For the two‐step qPCR assay, each reaction was prepared in a total volume of 25 μL containing 12.5 μL of HS Prime qPCR Premix (2×) (Genetbio, Republic of Korea) and 5 μL of synthesised cDNA as the template. For the one‐step RT‐qPCR assay, each reaction was prepared in a total volume of 25 μL containing 12.5 μL of SuPrimeScript qRT‐PCR Premix (2×), and with 5 μL of extracted RNA as the template. The final primer and probe concentrations were 900 and 250 nM, respectively, for both assays. Cycling conditions were initially tested at 95°C for 5 min, followed by 45 cycles of 95°C for 5 s and annealing at 56°C, 58°C, 60°C and 62°C for 10 s. Based on these trials, the optimised condition was set to 95°C for 10 s and 60°C for 20 s, which was applied to all subsequent assays.

#### 2.1.2 Construction of Plasmid DNA as a Positive Control

Plasmid DNA (pDNA) containing the full‐length G gene (Accession No. JQ666283.1) of the SVCV genotype Ia was custom‐synthesised by Bioneer Corporation (Republic of Korea) and used as a double‐stranded positive control for qPCR assays. The synthesised pDNA was diluted in DEPC‐treated distilled water and quantified using a nanodrop spectrophotometer. Based on the measured concentration, the plasmid copy number was calculated and serially diluted to appropriate working concentrations for subsequent analyses.

#### 2.1.3 Generation of Standard Curves and Analytical Sensitivity (LoD_95_ _%_) Determination

Standard curves were established using 10‐fold serial dilutions of pDNA ranging from 10^7^ to 10^1^ copies per 5 μL, with each dilution tested in triplicate. The amplification efficiency (*E*) of the qPCR assay was calculated using the cycle threshold (*C*_t_) slope method and expressed as *E* = (10^−1/slope^ **–** 1) × 100. Linearity was assessed by the coefficient of determination (*R* ^2^). To evaluate assay robustness, the coefficient of variation (%CV) and standard deviation (SD) were calculated from *C*_t_ values obtained for each replicate.

To estimate the 95% limit of detection (LoD_95%_), a series of twofold dilutions of pDNA ranging from 1000 to 1.95 copies per reaction was prepared, and 24 replicate qPCR reactions were performed for each dilution (Kralik and Ricchi 2017). Probit regression analysis was applied to calculate the LoD_95%_ and its 95% confidence interval (CI). The *C*_t_ cut‐off value was determined by substituting the lowest concentration within the 95% CI into the standard curve equation, thereby converting the estimated LoD_95%_ into a *C*_t_‐based threshold (Caraguel et al. 2011). *C*_t_ values above this threshold were considered unreliable, as they corresponded to amplification from extremely low target copy numbers.

To validate the estimated LoD_95%_, plasmid dilutions corresponding to 2× and 1× the LoD_95%_ (14 and 7 copies per reaction, respectively) were prepared and tested in 96 replicate qPCR reactions under identical conditions. The detection rate was calculated as the proportion of positive reactions among the 96 replicates.

#### 2.1.4 Assessment of Analytical Specificity and Cross‐Reactivity

To analyse the cross‐reactivity of the developed SVCV qPCR assay diagnostic assay, the following viruses were tested: SVCV (genotype Ia), VHSV (genotype IVa), Red sea bream iridovirus (RSIV; RSIV type), Infectious spleen and kidney necrosis virus (ISKNV; ISKNV type), Flounder iridovirus (FLIV; TRBIV type), Lymphocystis disease virus (LCDV) and Viral nervous necrosis virus (VNNV; BFNNV type).

For bacterial cross‐reactivity verification, the following bacteria were tested: *Vibrio harveyi* (FP8370), *V. ichthyoenteri* (FP8487), *V. ordalii* , *V. campbellii* , *V. alginolyticus* , *V. anguillarum* (TR14001NF), *Streptococcus iniae* (FP5228), *S. parauberis* (FP3287), *Photobacterium damselae* (FP4101), *Lactococcus garvieae* (FP5245), *Edwardsiella tarda* (FP5060) and *E. coli* (JM109).

### 2.2 Preparation of Infectious and Inactivated Virus, RNA Extraction and Cell Culture

EPC cells were cultured at 25°C in L‐15 medium (Thermo Fisher Scientific, USA) supplemented with 10% foetal bovine serum (FBS; Thermo Fisher Scientific, USA) and 1% antibiotic–antimycotic solution (100 U/mL penicillin, 100 μg/mL streptomycin and 25 μg/mL amphotericin B; Gibco). The cells were maintained until a confluent monolayer was formed, after which viral infection was conducted. Prior to viral inoculation, the culture medium was replaced with L‐15 containing 2% FBS. The SVCV (GenBank accession number: PX136979; genotype Ia) filtrate was prepared by homogenising infected common carp tissue in L‐15 medium, followed by centrifugation at 10,000× *g* for 5 min at 4°C and filtration through a 0.45 μm syringe filter. One millilitre of the filtrate was added to EPC cell monolayers. After 4 h of incubation, the inoculum was removed, the cells were washed once with fresh medium and then maintained in L‐15 at 25°C for 7 days. The resulting culture supernatant was collected and used as the viral inoculum for subsequent experiments.

To prepare the inactivated virus, 200 μL of viral stock was heated at 75°C for 3 min and treated with 1% SDS (Acros Organics, USA) with shaking at 200 rpm for 30 min at room temperature, followed by a 400‐fold dilution to reduce cytotoxicity.

To extract viral RNA, 200 μL of each infectious and inactivated sample was processed using the GeneAll Hybrid‐R Kit (GeneAll, Republic of Korea) according to the manufacturer's protocol.

To validate infectivity, 200 μL of each sample was inoculated onto EPC monolayers grown in 48‐well plates after 4 h of adsorption, the inoculum was removed, cells were washed, fresh L‐15 medium supplemented with 2% FBS was added, and the cultures were monitored daily for CPE for 7 days, while the diluted inactivated virus was tested in the same manner to assess residual infectivity and SDS cytotoxicity.

### 2.3 Development and Optimisation of the SVCV vqPCR Assay

#### 2.3.1 Intercalating Dye Assessment and Pretreatment Optimisation

Propidium monoazide (PMAxx) and platinum (IV) chloride (PtCl_4_) were selected as candidate nucleic acid–intercalating dyes to optimise the vqPCR assay. Infectious and inactivated SVCV suspensions (200 μL) were treated with PMAxx at concentrations of 10, 25, 50, 100 and 200 μM or PtCl_4_ at 50, 100, 250, 500, 1000, 2000 and 5000 μM. To enhance dye penetration efficiency, a parallel treatment group was prepared with 0.01% Triton X‐100 under identical conditions. All samples were wrapped with aluminium foil to maintain dark conditions and gently agitated at 200 rpm for 30 min at room temperature. Following incubation, PMAxx‐treated samples were photoactivated using a LED photolysis device (PMA‐Lite, Biotium, USA) for 30 min. In contrast, PtCl_4_‐treated samples did not undergo a photoactivation step, as it is not required for this reagent.

To determine the optimal reaction time that effectively suppresses signals from inactivated SVCV without compromising the detection of infectious virus, the vqPCR assay was performed using both infectious and inactivated viral samples with the previously determined optimal concentration (25 μM), followed by pretreatment (10, 20 and 30 min of shaking) and photoactivation (10, 20 and 30 min of irradiation). Based on these results, the final optimised condition (25 μM PMAxx, shaking for 30 min in the dark, followed by 20 min photoactivation) was applied. The suppression efficiency of inactivated virus signals was evaluated by calculating the delta *C*_t_ (*C*_t_ with PMAxx—*C*_t_ without PMAxx), and the selective detection of infectious virus was confirmed by comparison with vqPCR, qPCR and CPE results.

#### 2.3.2 Comparative Detection of Infectious and Inactivated SVCV by qPCR, vqPCR and CPE

The optimised vqPCR pretreatment (25 μM PMAxx treatment, followed by 30 min shaking in the dark and 20 min photoactivation) was applied to both infectious and heat‐inactivated SVCV. Viral samples were prepared as described in Section 2.2 and used in parallel for qPCR, vqPCR and cell culture–based CPE assays. For molecular assays (qPCR and vqPCR), 200 μL of the pretreated samples were immediately subjected to RNA extraction, while the remaining aliquots were inoculated onto EPC cell monolayers to assess infectivity through the observation of CPE.

### 2.4 Analytical Performance of vqPCR Across Defined Infectious‐Fraction Mixtures of SVCV

To evaluate whether the vqPCR assay can selectively detect infectious virus in samples containing mixtures of infectious and inactivated SVCV, its performance was compared with that of qPCR. Viral suspensions with defined proportions of infectious SVCV (0%, 1%, 10%, 30%, 50%, 70%, 90%, 99%, 99.9% and 100%) were prepared in 200 μL volumes. For each proportion, vqPCR and qPCR assays were performed in triplicate. In parallel, each sample was inoculated into EPC cells, and CPE development was monitored for 7 days.

### 2.5 Diagnostic Evaluation of qPCR and vqPCR Assays

#### 2.5.1 Collection of Clinical Samples and Experimental Infections

For the clinical evaluation of SVCV, a total of 500 common carp were collected from aquaculture farms in Jincheon, Republic of Korea (*n* = 500; total length: 16.5 ± 2.7 cm; weight: 108 ± 10.5 g) and transported to the laboratory. To secure negative control tissues, kidney and spleen tissues (30 mg each) were aseptically excised from 100 fish and stored at −80°C until use. The fish were confirmed to be SVCV‐negative by the WOAH‐recommended nested PCR assay (World Organisation for Animal Health (WOAH) 2023).

SVCV positive samples were obtained by experimentally infecting 150 common carp that had been acclimated at 15°C. Fish were intraperitoneally inoculated with either 10^6^ or 10^4^ SVCV copies/fish (*n* = 75 per group). At 1, 4, 7, 10 and 14 days post‐infection, 15 fish were randomly sampled from each group, and spleen and kidney tissues were aseptically excised for subsequent analyses.

All animal experiments were conducted in strict accordance with the Institutional Animal Care and Use Committee (IACUC) guidelines at Gyeongsang National University (Approval number: GNU‐250204‐E0022).

#### 2.5.2 Determination of Diagnostic Sensitivity (DSe) and Specificity (DSp)

For the evaluation of DSe and DSp, negative control tissues (*n* = 100) confirmed by the WOAH‐recommended nested PCR and positive samples obtained from experimentally infected common carp (*n* = 150, of which 100 fish were analysed) were used. Kidney and spleen tissues were homogenised in 1.5 mL of L‐15 medium supplemented with 2% FBS, filtered through 0.45 μm syringe filters, and divided for analysis by qPCR, vqPCR and cell culture.

The diagnostic performance of each method was evaluated using different reference standards: qPCR results were compared with those of the WOAH nested PCR assay, while vqPCR results were validated against EPC cell culture, in which CPE were monitored for 7 days.

#### 2.5.3 Repeatability

The repeatability of the vqPCR assay was evaluated using three concentrations of SVCV (10^8^, 10^6^ and 10^4^ SVCV copies per 200 μL). Experiments were conducted twice daily for 20 days, with two replicates per concentration, to assess within‐run, between‐run and between‐day variations. Repeatability was determined in accordance with Clinical and Laboratory Standards Institute (CLSI) guidelines (Tholen et al. 2004).

#### 2.5.4 Evaluation of Inter‐Laboratory Reproducibility

For reproducibility assessment, a total of 100 fish tissue samples (approximately 30 mg each; 50 SVCV‐positive and 50 SVCV‐negative) were randomly labelled and distributed to three independent research institutions. At each institution, one researcher independently conducted the experiments. Tissue samples were homogenised and nucleic acids were extracted as described in Section 2.2. Each sample was divided into three 200 μL aliquots: one was analysed by qPCR, another by vqPCR following PMAxx‐based viability pretreatment and the third by CPE analysis in EPC cells. Upon completion of all assays, *C*_t_ values from qPCR and vqPCR, as well as CPE results, were compared across the three laboratories to evaluate inter‐laboratory reproducibility.

### 2.6 Application of vqPCR to Environmental Freshwater (Laboratory Simulation)

To evaluate the applicability of the developed vqPCR assay in freshwater conditions, SVCV was spiked into both autoclaved freshwater and environmental freshwater used for laboratory‐based simulation at a final concentration of 1 × 10^8^ copies/mL. Environmental freshwater was prepared using tap water that had been dechlorinated to remove residual chlorine prior to the simulation. The water temperature was maintained at 15°C, and the pH was adjusted to 7. The inoculated water samples were incubated at 4°C, 15°C and 25°C, with aliquots collected on days 0, 1, 4, 7, 10, 14 and 30. At each time point, 200 μL of the sample was collected and filtered through a sterile syringe filter (0.45 μm pore size) to remove debris and particulate matter prior to nucleic acid extraction. Subsequently, 200 μL of the processed sample was analysed by qPCR, while another 200 μL was subjected to vqPCR to compare the reduction in viral loads over time. Of note, basic physicochemical parameters of the environmental freshwater were not systematically controlled or monitored in this study. Therefore, these results reflect laboratory‐simulated conditions rather than fully characterised natural aquatic environments.

### 2.7 Statistical Analysis

Statistical analyses were conducted using GraphPad Prism 9.5 (https://www.graphpad.com/scientific‐software/prism/). One‐way ANOVA followed by Tukey's multiple comparisons test was applied to evaluate *C*_t_ value differences according to PMAxx or PtCl_4_ concentrations, Triton X‐100 treatment, and pretreatment conditions including shaking and photoactivation.

Two‐way ANOVA followed by Tukey's test was used to compare *C*_t_ values between infectious and inactivated SVCV and to assess the effects of infectious SVCV proportion and PMAxx treatment. Delta *C*_t_ values (*C*_t_ with PMAxx—*C*_t_ without PMAxx) were analysed using one‐way ANOVA with Tukey's test.

One‐way ANOVA with Dunnett's correction was performed to analyse changes in SVCV viability in seawater over time, with each time point compared with the control. For reproducibility assessment, statistical differences among technicians were evaluated using two‐way ANOVA with Tukey's test.

Inter‐technician and inter‐laboratory agreement was further assessed using linear regression analysis, Pearson's correlation coefficient (*ρ*), and the concordance correlation coefficient (CCC) with 95% confidence intervals.

## 3 Results

### 3.1 Development of an SVCV qPCR Assay

#### 3.1.1 Amplification Efficiency and Analytical Sensitivity (LoD_95_ _%_) of the qPCR Assay

**FIGURE 1:**
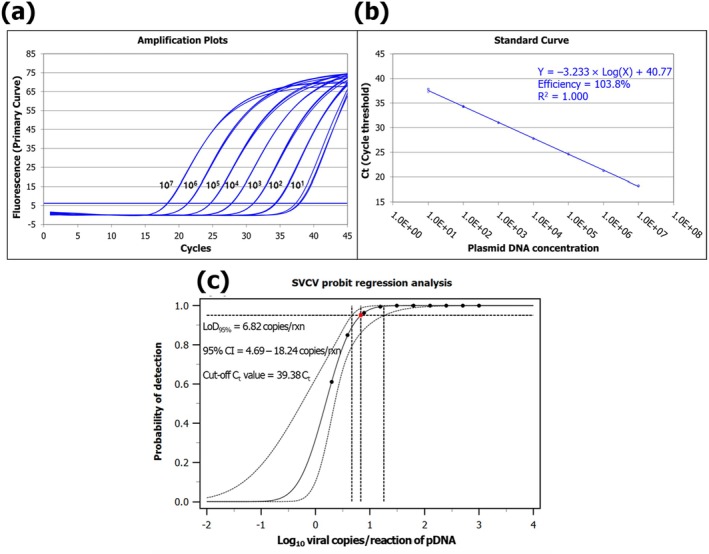
Amplification plots, standard curve, and probit regression analysis of spring viremia of carp virus (SVCV) quantitative PCR (qPCR). (a) Plasmid DNA was 10‐fold serially diluted from 10^7^ to 10^1^ copies/reaction and subjected to qPCR to generate amplification plots. (b) Standard curve of *C*_t_ vs. plasmid DNA copy number. (c) Low‐concentration plasmid DNA was tested in 24 replicate reactions, and probit regression analysis was performed to estimate the 95% limit of detection (LoD_95%_) and its confidence interval.

Primers and a hydrolysis probe targeting the glycoprotein (G) gene were designed based on phylogenetic analysis of 45 SVCV isolates (Table 1). The qPCR assay exhibited optimal amplification at an annealing/extension temperature of 60°C, using a thermal cycling program of 95°C for 5 min, followed by 45 cycles of 95°C for 10 s and 60°C for 20 s (Table S1). Ten‐fold serial dilutions of plasmid DNA containing the SVCV G gene (10^7^–10^1^ copies/reaction) generated amplification curves with clear separation between template concentrations (Figure 1a). The standard curve demonstrated a linear correlation between plasmid copy number and *C*_t_ values (*R* ^2^ = 1.000), with an amplification efficiency of 103.8% (slope = −3.233) (Figure 1b). Analytical sensitivity was assessed using 2‐fold serial dilutions of plasmid DNA (1000–1.95 copies/reaction), yielding an LoD_95%_ of 6.82 copies/reaction (95% CI: 4.69–18.24) (Table S2, Figure 1c). A diagnostic cut‐off of 39.38 *C*_t_ was established from the regression analysis, above which detection was considered unreliable. LoD_95%_ validation using plasmid DNA at 2× and 1× LoD_95%_ (14 and 7 copies/reaction) in 96 replicates showed detection rates of 100% (14 copies, 96/96) and 93.75% (7 copies, 90/96), with mean *C*_t_ values of 37.99 (SD 0.76, CV 1.99%) and 39.29 (SD 0.97, CV 2.47%), respectively (Table S3).

**TABLE 1:** List of primers and probes used in this study.

Name	Primer and probe	Length	*T*_m_ (°C)	GC%	Product size (bp)	Sequence (5′‐3′)
SVCV quantitative PCR	1351F	20	56.2	50	170	GTGGATGCAGTGGTAGAATG
	1520R	20	57.8	50		GACCGCATTTCGTGTGATTC
	1441P	25	66.1	52		[FAM]CTCATCAGGTGCTGTGTTGCTTGCA[BHQ1]

#### 3.1.2 Analytical Specificity and Cross‐Reactivity

To assess assay specificity, the qPCR was challenged with nucleic acids from 19 different aquatic pathogens. Notably, only SVCV showed positive results, indicating no cross‐reactivity (Table S4).

### 3.2 Development and Optimisation of an SVCV vqPCR Assay

#### 3.2.1 Viability Dye Selection and Assay Optimisation

**FIGURE 2:**
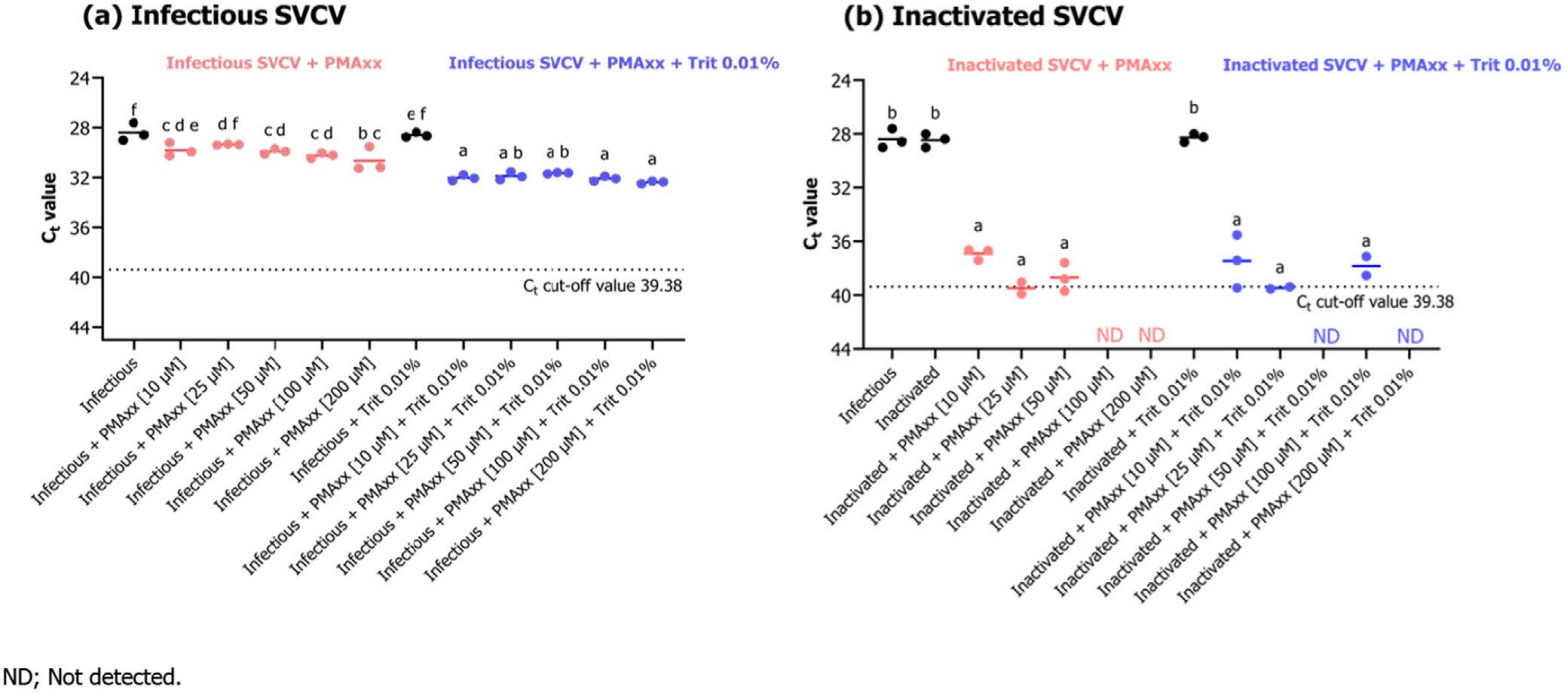
Performance evaluation of the viability quantitative PCR (vqPCR) assay for detecting inactivated and infectious spring viremia of carp virus (SVCV) using propidium monoazide (PMAxx) at concentrations of 10–200 μM, with or without 0.01% Triton X‐100. The black dotted line indicates the qPCR assay cut‐off cycle threshold (*C*_t_) value, and the solid line within each interval represents the mean *C*_t_ value (*n* = 3). One‐way ANOVA followed by Tukey's multiple comparisons test was performed within each experimental group to evaluate differences among treatment subgroups.

The effects of PMAxx and PtCl_4_, with or without 0.01% Triton X‐100, on the detection of infectious and inactivated SVCV were evaluated using the vqPCR assay. For infectious SVCV, amplification was maintained at PMAxx concentrations up to 25 μM, whereas the addition of Triton X‐100 inhibited amplification at all concentrations (Figure 2). In contrast, amplification of inactivated SVCV was efficiently suppressed at 25, 100 and 200 μM PMAxx, or at 25, 50 and 200 μM in the presence of Triton X‐100. However, PMAxx concentrations exceeding 25 μM or the use of Triton X‐100 also inhibited amplification of infectious SVCV.

For PtCl_4_, amplification of infectious SVCV was unaffected at concentrations up to 500 μM when used alone, or up to 100 μM in combination with Triton X‐100 (Figure S2). Suppression of inactivated SVCV was observed only at concentrations ≥ 1000 μM; however, these levels (≥ 500 μM alone, or ≥ 250 μM with Triton X‐100) also inhibited detection of infectious SVCV. Compared with PMAxx, PtCl_4_ exhibited weaker selective suppression of inactivated virus within the concentration range that did not inhibit infectious particles.

Taken together, 25 μM PMAxx without Triton X‐100 was identified as the optimal pretreatment condition for the SVCV vqPCR assay, as it maximised suppression of signals from inactivated virus while preserving detection of infectious particles. Consequently, PtCl_4_ was considered unsuitable for this application.

**FIGURE 3:**
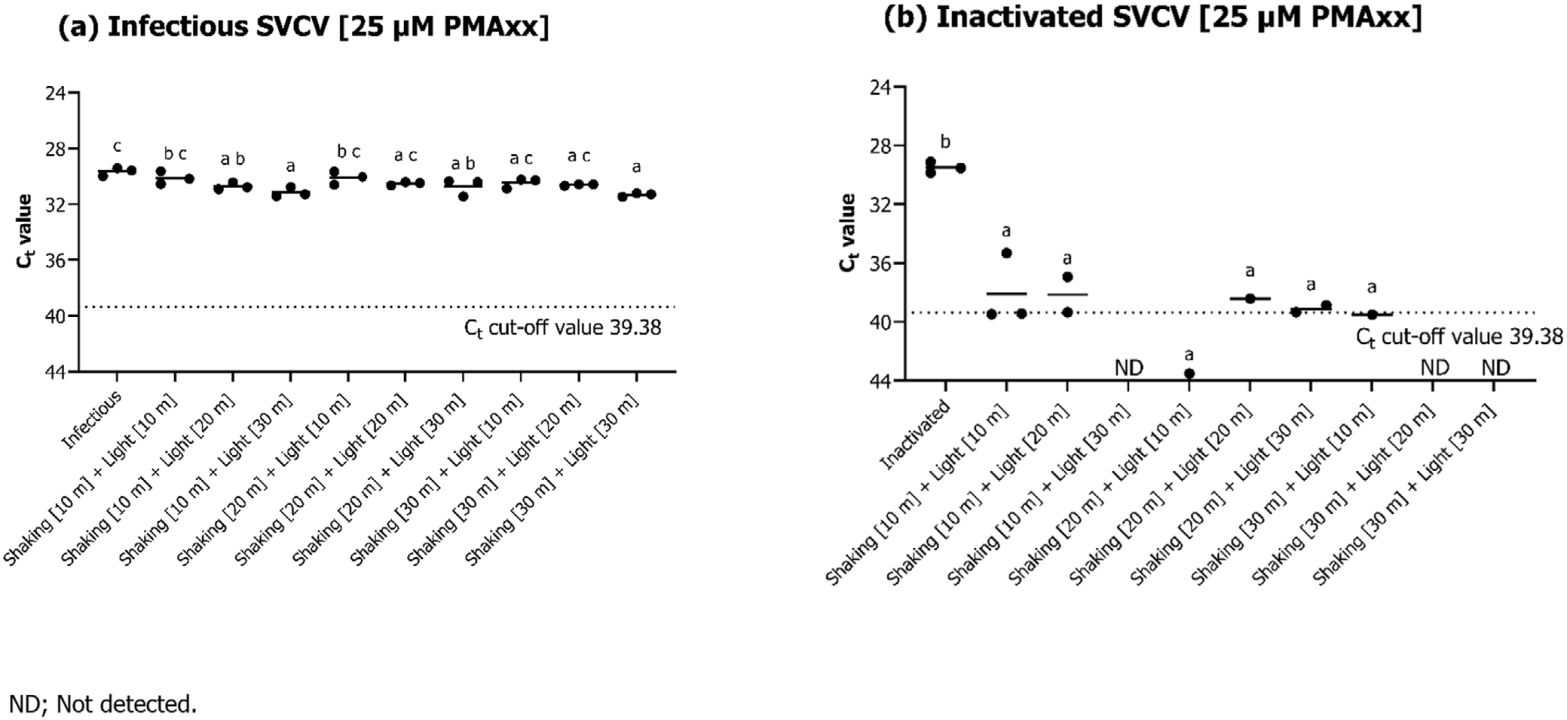
Viability quantitative PCR (vqPCR) assay was applied to optimise pretreatment conditions for discriminating infectious and inactivated spring viremia of carp virus (SVCV). Using the established optimal concentration of propidium monoazide (PMAxx, 25 μM), various combinations of shaking (10–30 min) and photoactivation (10–30 min) were evaluated (*n* = 3). The black dotted line represents the qPCR assay cut‐off cycle threshold (*C*_t_) value. Differences among treatment groups were analysed using one‐way ANOVA followed by Tukey's multiple comparisons test to determine statistical significance (*p* < 0.05). Bars labelled with different lowercase letters indicate statistically significant differences among groups.

To further optimise the pretreatment procedure, different shaking durations (10, 20 and 30 min) and photoactivation times (10, 20 and 30 min) were evaluated. The most effective conditions were incubation with shaking at 200 rpm for 30 min in the dark, followed by photoactivation for 20 min (Figure 3).

#### 3.2.2 Comparative Detection of Infectious and Inactivated SVCV by qPCR, vqPCR and CPE

**FIGURE 4:**
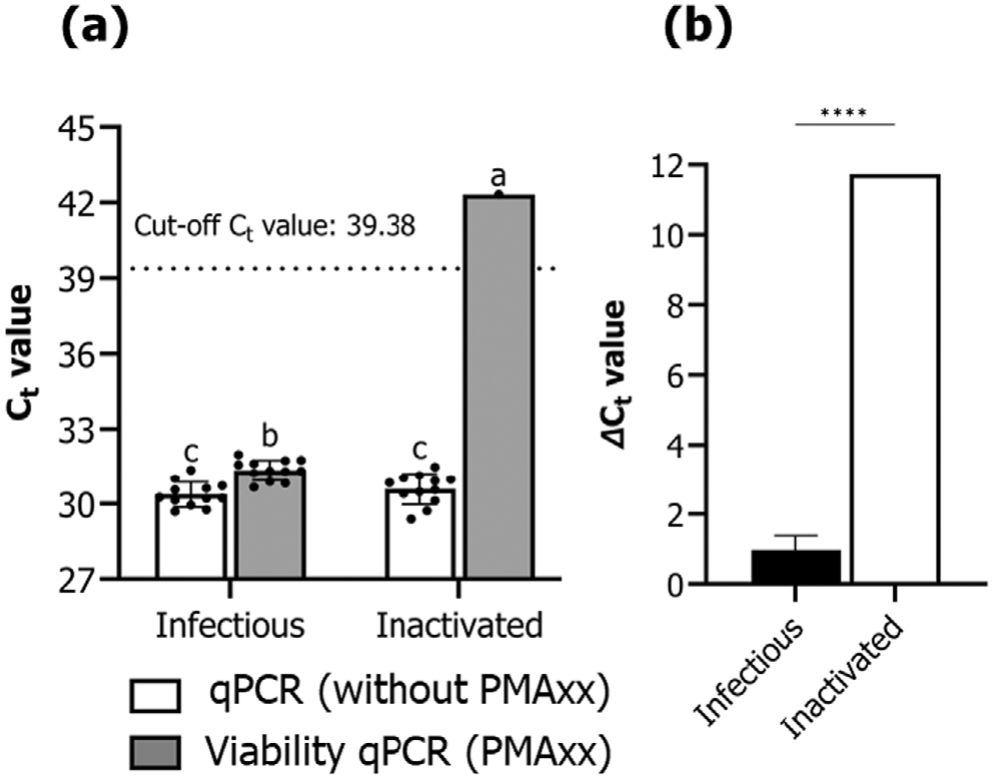
Evaluation of the performance of the viability quantitative PCR (vqPCR) assay for the selective detection of infectious and inactivated spring viremia of carp virus (SVCV) using propidium monoazide (PMAxx). (a) Comparison of cycle threshold (*C*_t_) values obtained from the qPCR assay (white bars) and vqPCR assay with PMAxx treatment (grey bars) for infectious and inactivated SVCV. Each bar represents the mean ± Standard deviation (SD) and individual points indicate replicate reactions (*n* = 12). Statistical analysis was performed using two‐way ANOVA followed by Tukey's multiple comparison test (*p* < 0.05). (b) Delta *C*_t_ values (*C*_t_ [with PMAxx] – *C*_t_ [without PMAxx]) for infectious and inactivated SVCV. Statistical differences were assessed using an unpaired *t*‐test. ****p < 0.0001.

The vqPCR assay with PMAxx treatment markedly reduced the detection of inactivated SVCV compared with the qPCR assay. For infectious SVCV, *C*_t_ values remained largely unchanged (delta *C*_t_ = 0.92), whereas inactivated SVCV showed a substantial increase in *C*_t_ values, with a mean delta *C*_t_ of 11.74. Only one of twelve replicates (8.3%) produced a detectable signal (*C*_t_ = 42.33), which exceeded the diagnostic cut‐off and was therefore considered outside the reliable detection range following PMAxx treatment (Figure 4). Consistently, all infectious SVCV samples produced clear CPE in EPC cells, whereas no CPE was observed in cells inoculated with inactivated SVCV (data not shown). These results confirm that the vqPCR assay selectively detects infectious SVCV by efficiently suppressing signals derived from inactivated virus (*p* < 0.0001).

### 3.3 vqPCR Performance Across Infectious‐Fraction Mixtures

**FIGURE 5:**
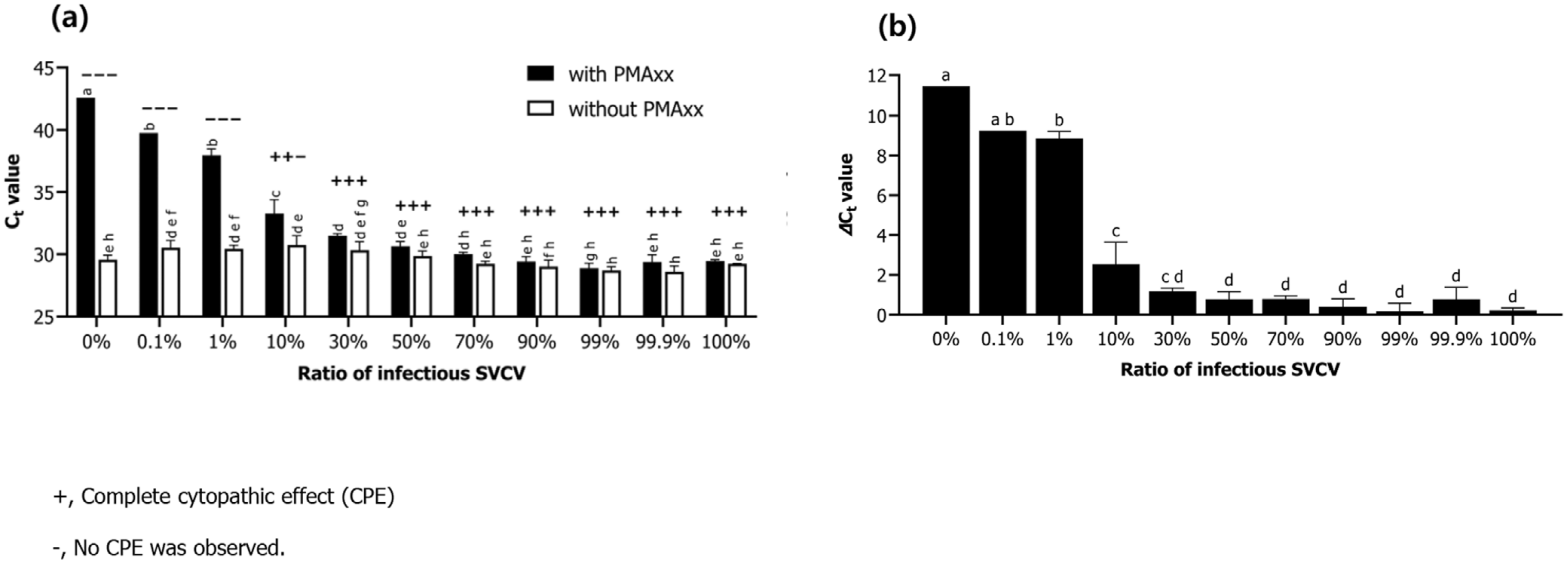
Viability quantitative PCR (vqPCR) assay results for the selective detection of infectious spring viremia of carp virus (SVCV) in samples containing mixtures of infectious and inactivated SVCV. qPCR assays were performed with or without propidium monoazide (PMAxx) treatment, depending on the proportion of infectious virus. (a) Cycle threshold (*C*_t_) values according to the proportion of infectious SVCV and the presence or absence of PMAxx. “+” and “–” indicate CPE‐positive and CPE‐negative results in cell culture, respectively. CPE evaluation was conducted in triplicate independent experiments. Statistical analysis was performed using two‐way ANOVA followed by Tukey's multiple comparisons test. (b) Delta *C*_t_ values according to the presence or absence of PMAxx, calculated as delta *C*_t_ = *C*_t_ (with PMAxx) − *C*_t_ (without PMAxx). Statistical differences were assessed using one‐way ANOVA with Tukey's multiple comparisons test. Bars labelled with different lowercase letters (a–h) are significantly different (*p* < 0.05) according to Tukey's multiple comparison test. Each bar represents the mean ± standard deviation (SD) from three independent experiments (*n* = 3).

Viability qPCR was conducted using mixtures of infectious and inactivated SVCV at various ratios (0%, 10%, 30%, 50%, 70%, 90%, 99%, 99.9% and 100%). In qPCR assays without PMAxx, *C*_t_ values showed negligible variation irrespective of the proportion of infectious SVCV (Figure 5). By contrast, in vqPCR assays with PMAxx treatment, *C*_t_ values progressively increased as the proportion of infectious virus decreased, with a delta *C*_t_ of approximately 3.83 between 100% and 10%. At 0% infectious SVCV, *C*_t_ values exceeded the diagnostic cut‐off (Figure 5). Consistently, CPE was absent when the proportion of infectious SVCV was ≤ 1%, corresponding to a vqPCR *C*_t_ value of ~37.96. Taken together, these results confirm that the vqPCR assay can reliably discriminate infectious from inactivated particles in a proportion‐dependent manner, in agreement with cell culture findings.

### 3.4 Diagnostic Evaluation of the vqPCR Assay

#### 3.4.1 DSe and DSp Compared With WOAH‐Recommended Assays

The WOAH nested PCR assay was performed on a subset of 100 infected and 100 non‐infected fish, yielding 100 positive and 100 negative results, respectively. The newly developed SVCV qPCR assay produced identical outcomes on the same samples, demonstrating complete concordance with the WOAH nested PCR (Table 2). Accordingly, both the DSe and DSp of the assay were calculated as 100%.

**TABLE 2:** Diagnostic sensitivity (DSe) and specificity (DSp) of the newly developed spring viremia of carp virus (SVCV) quantitative PCR (qPCR) compared with the WOAH nested PCR assay.

SVCV qPCR DSe and DSp		WOAH nested PCR assay		Total
		Positive	Negative	
SVCV quantitative PCR	Positive	100	0	100
	Negative	0	100	100
	Total	100	100	

Performance parameter	Value	95% CI
Sensitivity (%)	100	96.38 to 100
Specificity (%)	100	96.38 to 100
Positive predictive value (%)	100	96.38 to 100
Negative predictive value (%)	100	96.38 to 100
Accuracy (%)	100	98.17 to 100

For the vqPCR assay, diagnostic performance was assessed against the EPC cell culture. Inoculation of the same samples into EPC cells accurately distinguished all infected and non‐infected fish (Table 3). Among the 100 positive samples, the mean *C*_t_ values were 22.53 ± 4.01 for qPCR and 22.69 ± 3.88 for vqPCR, with a delta *C*_t_ of 0.16 ± 0.40. No significant difference in *C*_t_ values was observed between the two assays (*p* = 0.7726; Figure S3). For the sample yielding the highest vqPCR *C*_t_ value (36.61), mild CPE characterised by ~60% cell detachment was observed (data not shown). Thus, the vqPCR assay also achieved 100% DSe and 100% DSp relative to EPC cell culture.

**TABLE 3:** Diagnostic sensitivity (DSe) and specificity (DSp) of the spring viremia of carp virus (SVCV) viability quantitative PCR (vqPCR) assay compared with cell culture.

SVCV vqPCR DSe and DSp		SVCV CPE results		Total
		Positive	Negative	
SVCV viability quantitative PCR	Positive	100	0	100
	Negative	0	100	100
	Total	100	100	

Performance parameter	Value	95% CI
Sensitivity (%)	100	96.38 to 100
Specificity (%)	100	96.38 to 100
Positive predictive value (%)	100	96.38 to 100
Negative predictive value (%)	100	96.38 to 100
Accuracy (%)	100	98.17 to 100

#### 3.4.2 Repeatability Analysis of the vqPCR Assay

The repeatability of the SVCV vqPCR assay was assessed using three viral concentrations (10^8^, 10^6^ and 10^4^ SVCV copies per 200 μL). Experiments were conducted twice daily for 20 consecutive days, with duplicate samples at each concentration, to evaluate intra‐assay, inter‐assay and inter‐day variability (Table 4). The CVs for intra‐assay reproducibility ranged from 0.01% to 0.53%, those for inter‐assay reproducibility from 0.01% to 0.03%, and those for inter‐day reproducibility from 0.01% to 0.02% across the tested concentrations. The greatest variability was observed in intra‐assay measurements at the highest concentration (0.53%), whereas all other CVs remained below 0.1%. These findings demonstrate that the SVCV vqPCR assay possesses excellent precision and repeatability across a wide dynamic range of template concentrations.

**TABLE 4:** Repeatability evaluation of the viability quantitative PCR (vqPCR) assay over 20 days using high, medium, and low copy spring viremia of carp virus (SVCV) samples.

Assay	Category	SVCV viral copies/200 μL		
		High copy	Medium copy	Low copy
Within‐run	Mean *C*_t_ ± SD	18.38 ± 0.1	25.29 ± 0.0	30.52 ± 0.03
	CV (%)	0.53	0.01	0.1
Between‐run	Mean *C*_t_ ± SD	18.38 ± 0.01	25.29 ± 0.1	30.52 ± 0.0
	CV (%)	0.03	0.03	0.01
Between‐day	Mean *C*_t_ ± SD	18.38 ± 0.09	25.29 ± 0.18	30.52 ± 0.47
	CV (%)	0.01	0.01	0.02

#### 3.4.3 Reproducibility Evaluation of vqPCR Assay

Independent evaluations by three technicians (A–C) yielded highly consistent outcomes between the qPCR and vqPCR assays. All 50 SVCV‐positive samples were correctly identified by both assays, and no false positives were detected among negative samples. The mean C_t_ values were comparable between qPCR and vqPCR for each technician (Technician A: 21.57 ± 3.19 vs. 21.95 ± 3.14; Technician B: 20.97 ± 3.02 vs. 21.37 ± 3.04; Technician C: 21.47 ± 3.20 vs. 21.47 ± 3.15), with no statistically significant differences observed (Figure S4).

**FIGURE 6:**
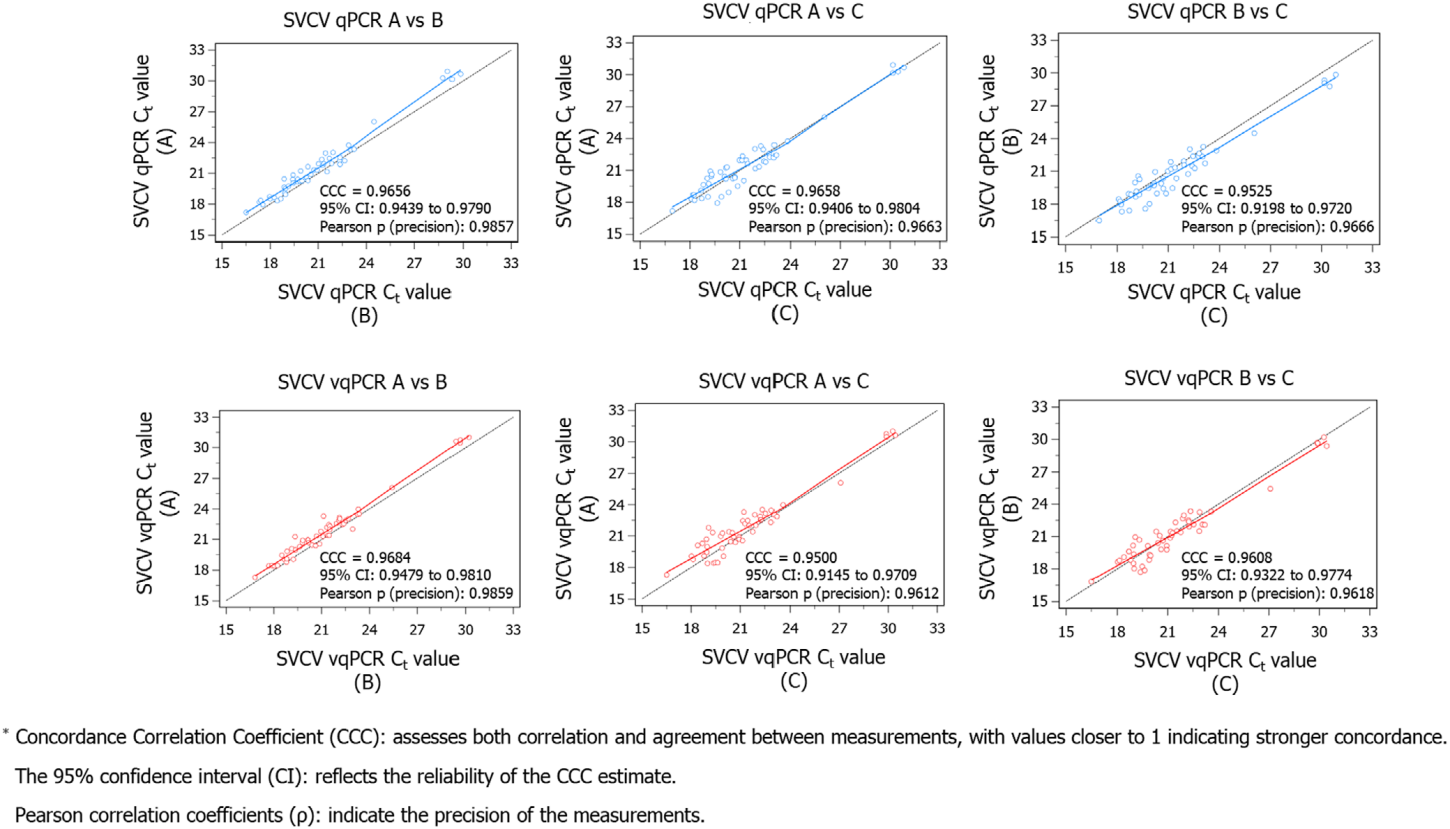
To evaluate the reproducibility of the diagnostic method, the agreement across different laboratories and technicians was assessed. Each panel shows linear regression and concordance correlation coefficient (CCC)* analysis comparing *C*_t_ values among three technicians (A–C).

Pairwise comparisons of *C*_t_ values across technicians demonstrated strong reproducibility for both qPCR and vqPCR assays (Figure 6). For qPCR, the concordance correlation coefficients (CCC) for all pairs (A–B, A–C and B–C) exceeded 0.95, with highly significant Pearson correlations (*p* < 0.0001), confirming strong concordance. Similarly, vqPCR also produced CCC values above 0.95 with significant Pearson correlations, indicating excellent inter‐operator agreement. In both assays, *C*_t_ values clustered tightly along the regression line and approximated the line of identity, suggesting minimal operator‐to‐operator variation and no systematic bias.

### 3.5 Application of the vqPCR Assay to a Naturally Inactivated Virus

**FIGURE 7:**
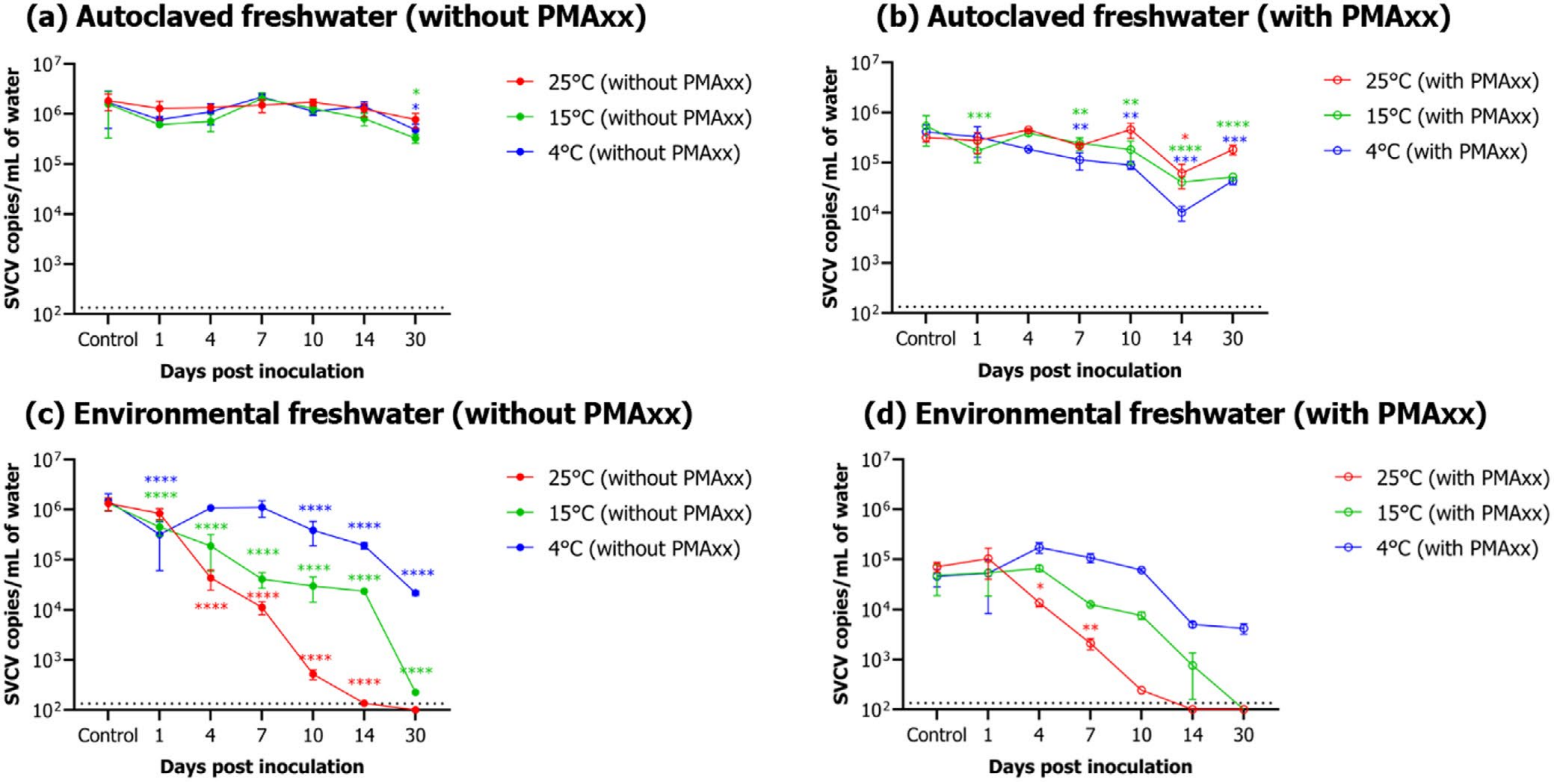
Changes in spring viremia of carp virus (SVCV) copy numbers over time in autoclaved and environmental freshwater, with or without propidium monoazide (PMAxx) treatment. (a) SVCV in autoclaved freshwater without PMAxx, (b) SVCV in autoclaved freshwater with PMAxx, (c) SVCV in environmental freshwater without PMAxx, and (d) SVCV in environmental freshwater with PMAxx. Error bars represent the mean ± standard deviation (*n* = 3). The dotted line indicates the detection limit. Statistical analysis was performed using one‐way ANOVA followed by Dunnett's multiple comparisons test, with each time point compared with the control. Asterisks denote statistically significant differences relative to the control (*p < 0.05, **p < 0.01, ***p < 0.001, ****p < 0.0001).

In autoclaved water, SVCV remained detectable for up to 14 days irrespective of temperature (Figure 7a,b). In the absence of PMAxx, the initial viral load was approximately 10^6^ copies/mL on day 0 and remained at comparable levels (10^5^–10^6^ copies/mL) at 4°C, 15°C and 25°C on day 14, indicating little change over time. In contrast, with PMAxx treatment, a gradual decline was observed, particularly at lower temperatures: at 4°C and 15°C, viral loads decreased to approximately 10^3^ and 10^4^ copies/mL, respectively, by day 14, while at 25°C, the virus fell below the detection limit during the same period (Figure 7b).

In environmental freshwater, SVCV persistence was clearly temperature‐dependent (Figure 7c,d). Without PMAxx, viral loads decreased progressively, with more rapid reductions at higher temperatures. At 25°C, SVCV dropped below the detection limit by day 14, while at 15°C levels remained around 10^4^–10^5^ copies/mL, and at 4°C about 10^5^–10^6^ copies/mL. With PMAxx treatment, the decline was accelerated across all conditions. At 25°C, viral loads fell below the detection limit by day 14. At 15°C, levels decreased to 10^3^ copies/mL, while at 4°C, they remained at 10^3^–10^4^ copies/mL by the end of the experiment.

## 4 Discussion

Various RT‐qPCR assays have been developed for SVCV detection (Yue et al. 2008; Zhang et al. 2009); however, due to limited inter‐laboratory validation and insufficient diagnostic reliability, none have been officially endorsed by WOAH. Therefore, the development of a WOAH‐compliant diagnostic method remains necessary. In accordance with WOAH‐recommended diagnostic guidelines, we developed a qPCR assay and conducted a full analytical and diagnostic validation covering analytical sensitivity (LoD), analytical specificity, diagnostic sensitivity, diagnostic specificity, repeatability and inter‐laboratory reproducibility through stage 1–3 performance evaluations, as outlined in the WOAH Aquatic Animal Diagnostic Test Manual (World Organisation for Animal Health (WOAH) 2019). Furthermore, a vqPCR assay was established by integrating a nucleic acid–intercalating viability dye to differentiate infectious from non‐infectious viral particles by selectively eliminating amplification signals from damaged or non‐viable virions.

The qPCR assay targeted the SVCV glycoprotein (G) gene, which shows sequence variability among geographically distinct isolates and is therefore relevant for epidemiological investigations (Stone et al. 2003). A 170 bp amplicon was designed considering the optimal qPCR assay amplicon range of 150–200 bp (Van Holm et al. 2021). The assay showed perfect linearity (*R* ^2^ = 1.000) and an amplification efficiency of 103.8% (slope = −3.233), meeting the recommended efficiency range of 95%–105% (Bustin and Huggett 2017). In the vqPCR assay, amplicon length represents a trade‐off between amplification efficiency and discrimination of infectious versus non‐infectious particles (delta *C*_t_). Van Holm et al. (2021) demonstrated that delta *C*_t_ increases markedly up to approximately 200 bp while maintaining acceptable efficiency, whereas further extension beyond 400 bp results in efficiency loss with limited gains in discrimination. Based on these findings and our data, the 170 bp target used in this study represents a balanced compromise, providing sufficient sensitivity for low‐titre samples while maintaining discriminatory power in the vqPCR assay. Nevertheless, evaluation of auxiliary targets within the 200–250 bp range may be useful for specific matrices such as environmental water or tissue samples, with careful consideration of potential reductions in efficiency and LoD. The qPCR assay developed in this study achieved a LoD_95%_ of 6.82 copies/reaction and a diagnostic cut‐off of 39.38 cycles, exceeding the sensitivity of previously reported SVCV assays (Yue et al. 2008). Conventional RT‐PCR assay, in contrast, is generally limited to detecting ≥ 10^3^ copies (Yue et al. 2008). Specificity testing against 19 aquatic pathogens showed no cross‐reactivity, confirming high analytical specificity and minimising false‐positive detection.

As vqPCR assay selectively amplifies viral genomes protected by intact envelopes or capsids (Leifels et al. 2021), an appropriate pretreatment step is essential. In this study, a capsid integrity–based inactivation protocol using 1% SDS (75°C for 3 min) was optimised, improving upon previously reported SDS‐NaOH conditions (Zhu et al. 2025). Among the tested reagents, PMAxx most effectively suppressed amplification from inactivated particles while preserving signals from infectious viruses, whereas PtCl_4_ inhibited infectious particles as well, consistent with concerns regarding its toxicity and limited applicability (Egorova and Ananikov 2017; Fraisse et al. 2018; Puente et al. 2020). Triton X‐100 also reduced amplification of infectious particles, as previously noted in some systems (Coudray‐Meunier et al. 2013; Fraisse et al. 2018). These findings underscore that the fundamental operating principle of vqPCR assay is strongly dependent on capsid integrity, highlighting that the physicochemical properties and reaction conditions of pretreatment reagents directly influence the assay's capacity to discriminate between infectious and non‐infectious viral particles. Notably, PMAxx most effectively fulfils its intended function by selectively binding to damaged capsids and suppressing amplification exclusively from non‐infectious particles. In contrast, PtCl_4_ and Triton X‐100 may impair the detection of infectious virions due to their potential to further disrupt capsid structures or exert nonspecific cytotoxic or chemically reactive effects.

Optimisation of viability dye concentration, incubation time and temperature is critical for vqPCR assay performance (Gao et al. 2021). The final pretreatment protocol (30 min dark incubation with shaking and 20 min photoactivation) was consistent with previous studies on enteric viruses and environmental samples (Randazzo et al. 2016; Thilakarathna et al. 2022). Under these conditions, PMAxx‐based vqPCR assay markedly reduced signals from inactivated SVCV while leaving detection of infectious particles nearly unaffected (delta *C*_t_ = 0.92). In mixed‐virus samples, *C*_t_ values increased proportionally with decreasing proportions of infectious virus, with an approximately 3.83 cycle shift between 100% and 10% infectious samples, consistent with the theoretical expectation for a 10‐fold dilution (Thermo Fisher Scientific n.d.). In mixed samples containing 1% infectious SVCV, CPE was no longer observed in cell culture, whereas vqPCR assay still detected a signal at approximately 37.96 *C*_t_. This suggests that the detection limit of the cell culture assay lies near this threshold and indicates that vqPCR assay is capable of detecting very low levels of infectious particles that fall below the sensitivity of cell culture. A similar trend has been reported for RSIV, in which vqPCR assay signals correlate well with TCID_50_ measurements (Jeong et al. 2025).

Both qPCR and vqPCR assays demonstrated 100% diagnostic sensitivity and specificity when compared with the WOAH‐recommended nested PCR (Stone et al. 2003) and EPC cell culture assays (Batts and Winton 1989; Wang et al. 2016), confirming the high reliability of both methods for determining infection status. The delta *C*_t_ difference between the two assays was minimal and not statistically significant (0.16 ± 0.40; *p* = 0.7726), clearly indicating that PMAxx treatment does not interfere with the detection of infectious virus. In addition, the observation of weak CPE at a *C*_t_ value of 36.61 indicates that the detection limit of the cell culture assay lies near this threshold and suggests that infectious virus may persist even in high‐*C*_t_ samples. Therefore, in the detection‐limit range, qPCR assay alone may not reliably determine infectivity, and vqPCR assay provides a valuable complementary tool capable of resolving this uncertainty.

The vqPCR assay also demonstrated excellent repeatability and reproducibility. According to MIQE 2.0 guidelines, intra‐assay CV values should remain below 15% and inter‐assay CV values below 25% (Bustin et al. 2025). In this study, intra‐ and inter‐assay CVs consistently remained well below these thresholds, even at low template concentrations. This is particularly notable given that qPCR assay performance at low copy numbers is known to be sensitive to variability arising from PCR assay inefficiencies and pipetting errors. The high CCC and Pearson correlation coefficients further confirmed strong inter‐operator reproducibility, consistent with previous reports (Daranas et al. 2018; Van Holm et al. 2021). However, inter‐platform reproducibility was not addressed in this study, and this aspect should be evaluated in future work to assess the broader applicability of the assay (Taylor et al. 2019; Hays et al. 2024).

The freshwater experiments in this study provide preliminary evidence for the potential applicability of vqPCR in detecting SVCV. However, as these tests were conducted under controlled laboratory‐simulated conditions, they may not fully capture the inherent complexity of natural aquatic environments. In real‐world systems, diverse environmental factors—such as dissolved oxygen (DO), pH, organic matter and turbidity—can influence PCR efficiency and PMAxx penetration (Linke et al. 2021; Chen 2025). Although these parameters were not systematically characterised in this study, previous research indicates that optimisation of PMAxx concentration and photoactivation conditions can effectively mitigate interference from complex matrices, such as those with high organic content (Randazzo et al. 2016; Chen 2025). Therefore, while our results provide a foundational baseline, further field‐based validation remains essential to confirm the robustness of this method.

In the environmental freshwater used here, microbial protease activity likely facilitated capsid degradation, leading to a temperature‐dependent decline in both SVCV infectivity and residual RNA (Ward et al. 1986). In contrast, in autoclaved freshwater, viral RNA persisted at approximately 10^5^–10^6^ copies/mL for up to 14 days despite the loss of infectivity. This underscores a fundamental limitation of standard qPCR, which can overestimate infection risk by detecting stable viral genomes regardless of particle integrity (Zeng et al. 2023). Notably, our vqPCR assay selectively suppressed signals from non‐infectious particles with compromised capsids, reflecting the loss of infectivity more sensitively than qPCR.

Furthermore, water‐based virus detection offers significant practical advantages, such as reduced handling stress on fish and the feasibility of population‐level surveillance. However, the persistence of viral RNA in water even after infectivity has declined can complicate the interpretation of standard qPCR results (Canh et al. 2022). The divergence between qPCR and vqPCR signals observed in this study suggests that vqPCR provides a more realistic reflection of infectious virus presence. Consequently, while this study establishes a foundational framework, further research incorporating diverse environmental parameters is required to fully validate this approach for field‐scale disease surveillance.

In conclusion, this study developed and analytically evaluated a vqPCR assay targeting the glycoprotein (G) gene of SVCV to improve the discrimination between infectious and non‐infectious viral particles. The assay demonstrated high analytical sensitivity, specificity and overall performance, reflecting infectivity loss more effectively than standard qPCR under controlled laboratory conditions. The vqPCR results were generally consistent with cell culture and WOAH‐recommended nested PCR, supporting its potential diagnostic utility. However, as this study was conducted under laboratory‐simulated conditions, further validation using diverse environmental samples and diagnostic settings is essential. Within these limitations, our findings suggest that the vqPCR approach may serve as a valuable complementary tool for monitoring infectious SVCV, potentially contributing to improved disease surveillance in freshwater aquaculture.

## Author Contributions

**Ha‐Jeong Son:** conceptualisation, methodology, validation, formal analysis, investigation, data curation, visualisation, writing – original draft, writing – review and editing. **Min‐Young Sohn:** conceptualisation, investigation, formal analysis. **Jae‐Ok Kim:** supervision, project administration, funding acquisition. **Hee Jung Choi:** supervision, project administration, funding acquisition. **Mun‐Gyeong Kwon:** supervision, project administration, funding acquisition. **Jeong‐Tae Lee:** investigation, formal analysis. **Gyoungsik Kang:** conceptualisation, methodology, validation, formal analysis, investigation. **HyeongJin Roh:** conceptualisation, methodology, validation, formal analysis, investigation. **Chan‐Il Park:** conceptualisation, funding acquisition, data curation, supervision, resources, writing – review and editing. **Kyung‐Ho Kim:** conceptualisation, methodology, validation, formal analysis, investigation, data curation, visualisation, writing – original draft, writing – review and editing, funding acquisition, supervision, resources.

## Funding

This work was supported by National Fishery Products Quality Management Service, NFQS2026001.

## Conflicts of Interest

The authors declare no conflicts of interest.

## Supporting information


**Figures S1‐S4:** jfd70163‐sup‐0001‐Supinfo.pptx.

**Table S1:** Performance evaluation of the spring viremia of carp virus (SVCV) quantitative PCR (qPCR) assay diagnostic assay at different annealing‐extension temperatures.
**Table S2:** Limit of detection at 95% (LoD95%) of the spring viremia of carp virus (SVCV) quantitative PCR (qPCR) assay at different plasmid DNA (pDNA) concentrations.
**Table S3:** Verification of the detection probability at 2× and 1× the 95% limit of detection (LoD95%) for the spring viremia of carp virus (SVCV) quantitative PCR (qPCR) assay.
**Table S4:** Cross‐reactivity of the Spring viraemia of carp virus (SVCV) quantitative PCR (qPCR) assay with various aquatic pathogens.

## Data Availability

The data that support the findings of this study are available from the corresponding author upon reasonable request.

## References

[jfd70163-bib-0001] Ahne, W. , H. V. Bjorklund , S. Essbauer , N. Fijan , G. Kurath , and J. R. Winton . 2002. “Spring Viremia of Carp (SVC).” Diseases of Aquatic Organisms 52: 261–272. 12553453 10.3354/dao052261

[jfd70163-bib-0002] Ashraf, U. , Y. Lu , L. Lin , J. Yuan , M. Wang , and X. Liu . 2016. “Spring Viraemia of Carp Virus: Recent Advances.” Journal of General Virology 97, no. 5: 1037–1051. 10.1099/jgv.0.000436. 26905065

[jfd70163-bib-0003] Batts, W. N. , and J. R. Winton . 1989. “Enhanced Detection of Infectious Hematopoietic Necrosis Virus and Other Fish Viruses by Pretreatment of Cell Monolayers With Polyethylene Glycol.” Journal of Aquatic Animal Health 1, no. 4: 284–290.

[jfd70163-bib-0004] Braunstein, G. D. , L. Schwartz , P. Hymel , and J. Fielding . 2021. “False Positive Results With SARS‐CoV‐2 RT‐PCR Tests and How to Evaluate a RT‐PCR‐Positive Test for the Possibility of a False Positive Result.” Journal of Occupational and Environmental Medicine 63, no. 3: e159–e162. 33405498 10.1097/JOM.0000000000002138 PMC7934325

[jfd70163-bib-0005] Bustin, S. , and J. Huggett . 2017. “qPCR Primer Design Revisited.” Biomolecular Detection and Quantification 14: 19–28. 29201647 10.1016/j.bdq.2017.11.001 PMC5702850

[jfd70163-bib-0007] Bustin, S. A. , J. M. Ruijter , M. J. B. van den Hoff , et al. 2025. “MIQE 2.0: Revision of the Minimum Information for Publication of Quantitative Real‐Time PCR Experiments Guidelines.” Clinical Chemistry 71, no. 6: 634–651. 40272429 10.1093/clinchem/hvaf043

[jfd70163-bib-0008] Canh, V. D. , M. Liu , J. Sangsanon , and H. Katayama . 2022. “Capsid Integrity Detection of Pathogenic Viruses in Waters: Recent Progress and Potential Future Applications.” Science of the Total Environment 827: 154258. 35248642 10.1016/j.scitotenv.2022.154258

[jfd70163-bib-0048] Caraguel, C. G. , H. Stryhn , N. Gagné , I. R. Dohoo , and K. L. Hammell . 2011. “Selection of a Cutoff Value for Real‐Time Polymerase Chain Reaction Results to Fit a Diagnostic Purpose: Analytical and Epidemiologic Approaches.” Journal of Veterinary Diagnostic Investigation 23, no. 1: 2–15. 10.1177/104063871102300102. 21217022

[jfd70163-bib-0009] Chen, Z. 2025. “Enhanced Detection of Viable *Escherichia coli* O157:H7 in Romaine Lettuce Wash Water Using On‐Filter Propidium Monoazide‐Quantitative PCR.” Microorganisms 13: 34. 10.3390/microorganisms13010034 PMC11767674 39858802

[jfd70163-bib-0010] Chen, Z. Y. , H. Liu , Z. Q. Li , and Q. Y. Zhang . 2008. “Development and Characterization of Monoclonal Antibodies to Spring Viraemia of Carp Virus.” Veterinary Immunology and Immunopathology 123: 266–276. 18378003 10.1016/j.vetimm.2008.02.011

[jfd70163-bib-0011] Coudray‐Meunier, C. , A. Fraisse , S. Martin‐Latil , L. Guillier , and S. Perelle . 2013. “Discrimination of Infectious Hepatitis a Virus and Rotavirus by Combining Dyes and Surfactants With RT‐qPCR.” BMC Microbiology 13: 216. 10.1186/1471-2180-13-216. 24083486 PMC3853579

[jfd70163-bib-0012] Daranas, N. , A. Bonaterra , J. Francés , J. Cabrefiga , E. Montesinos , and E. Badosa . 2018. “Monitoring Viable Cells of the Biological Control Agent *Lactobacillus plantarum* PM411 in Aerial Plant Surfaces by Means of a Strain‐Specific Viability Quantitative PCR Method.” Applied and Environmental Microbiology 84: e00107–e00118. 29523544 10.1128/AEM.00107-18 PMC5930365

[jfd70163-bib-0013] Egorova, K. S. , and V. P. Ananikov . 2017. “Toxicity of Metal Compounds: Knowledge and Myths.” Organometallics 36, no. 21: 4071–4090.

[jfd70163-bib-0014] Fijan, N. N. 1973. “Infectious Dropsy in Carp a Disease Complex.” In Diseases of Fish, edited by L. E. Mawdesley‐Thomas , 39–51. Academic Press.

[jfd70163-bib-0015] Fittipaldi, N. , M. Segura , D. Grenier , and M. Gottschalk . 2012. “Virulence Factors Involved in the Pathogenesis of the Infection Caused by the Swine Pathogen and Zoonotic Agent *Streptococcus suis* .” Future Microbiology 7, no. 2: 259–279. 22324994 10.2217/fmb.11.149

[jfd70163-bib-0016] Fraisse, A. , F. Niveau , C. Hennechart‐Collette , C. Coudray‐Meunier , S. Martin‐Latil , and S. Perelle . 2018. “Discrimination of Infectious and Heat‐Treated Norovirus by Combining Platinum Compounds and Real‐Time RT‐PCR.” International Journal of Food Microbiology 269: 64–74. 10.1016/j.ijfoodmicro.2018.01.015. 29421360

[jfd70163-bib-0017] Gao, S. , C. Sun , H. Hong , R. Gooneratne , A. Mutukumira , and X. Wu . 2021. “Rapid Detection of Viable *Cronobacter sakazakii* in Powdered Infant Formula Using Improved Propidium Monoazide (PMAxx) and Quantitative Recombinase Polymerase Amplification (qRPA) Assay.” Food Control 124: 107899.

[jfd70163-bib-0018] Gardner, S. N. , and T. Slezak . 2014. “Simulate_PCR for Amplicon Prediction and Annotation From Multiplex, Degenerate Primers and Probes.” BMC Bioinformatics 15: 237. 25005023 10.1186/1471-2105-15-237 PMC4226945

[jfd70163-bib-0019] Hays, A. , M. Wissel , K. Colletti , R. Soon , M. Azadeh , and J. Smith . 2024. “Recommendations for Method Development and Validation of qPCR and dPCR Assays in Support of Cell and Gene Therapy Drug Development.” AAPS Journal 26: 24. 38316745 10.1208/s12248-023-00880-9

[jfd70163-bib-0020] Jeong, J. M. , G. Kang , J. O. Kim , J. T. Lee , C. I. Park , and K. H. Kim . 2025. “Red Sea Bream Iridovirus Stability in Freeze–Thaw Cycles: Quantitative Assays of Infectious Particles.” Animals 15, no. 12: 1699. 40564252 10.3390/ani15121699 PMC12189574

[jfd70163-bib-0021] Kim, K. H. , G. Kang , W. S. Woo , M. Y. Sohn , H. J. Son , and C. I. Park . 2023. “Development of a Propidium Monoazide‐Based Viability Quantitative PCR Assay for Red Sea Bream Iridovirus Detection.” International Journal of Molecular Sciences 24: 3426. 36834834 10.3390/ijms24043426 PMC9958570

[jfd70163-bib-0047] Kralik, P. , and M. Ricchi . 2017. “A Basic Guide to Real‐Time PCR in Microbial Diagnostics: Definitions, Parameters, and Everything.” Frontiers in Microbiology 8: 108. 10.3389/fmicb.2017.00108. 28210243 PMC5288344

[jfd70163-bib-0022] Leifels, M. , D. Cheng , E. Sozzi , et al. 2021. “Capsid Integrity Quantitative PCR to Determine Virus Infectivity in Environmental and Food Applications—A Systematic Review.” Water Research X 11: 100080. 33490943 10.1016/j.wroa.2020.100080 PMC7811166

[jfd70163-bib-0023] Linke, R. B. , S. Zeki , R. Mayer , et al. 2021. “Identifying Inorganic Turbidity in Water Samples as Potential Loss Factor During Nucleic Acid Extraction: Implications for Molecular Fecal Pollution Diagnostics and Source Tracking.” Frontiers in Microbiology 12: 660566. 34745021 10.3389/fmicb.2021.660566 PMC8565874

[jfd70163-bib-0024] Nocker, A. , and A. K. Camper . 2009. “Novel Approaches Toward Preferential Detection of Viable Cells Using Nucleic Acid Amplification Techniques.” FEMS Microbiology Letters 291, no. 2: 137–142. 19054073 10.1111/j.1574-6968.2008.01429.x

[jfd70163-bib-0025] Padhi, A. , and B. Verghese . 2012. “Molecular Evolutionary and Epidemiological Dynamics of a Highly Pathogenic Fish Rhabdovirus, the Spring Viremia of Carp Virus (SVCV).” Veterinary Microbiology 156, no. 1–2: 54–63. 22033044 10.1016/j.vetmic.2011.10.005

[jfd70163-bib-0026] Puente, H. , W. Randazzo , I. Falcó , A. Carvajal , and G. Sánchez . 2020. “Rapid Selective Detection of Potentially Infectious Porcine Epidemic Diarrhea Coronavirus Exposed to Heat Treatments Using Viability RT‐qPCR.” Frontiers in Microbiology 11: 1911. 32973701 10.3389/fmicb.2020.01911 PMC7472829

[jfd70163-bib-0027] Rahman, M. T. , M. S. Uddin , R. Sultana , A. Moue , and M. Setu . 2013. “Polymerase Chain Reaction (PCR): A Short Review.” Anwer Khan Modern Medical College Journal 4, no. 1: 30–36.

[jfd70163-bib-0028] Randazzo, W. , F. López‐Gálvez , A. Allende , R. Aznar , and G. Sánchez . 2016. “Evaluation of Viability PCR Performance for Assessing Norovirus Infectivity in Fresh‐Cut Vegetables and Irrigation Water.” International Journal of Food Microbiology 229: 1–6. 27085970 10.1016/j.ijfoodmicro.2016.04.010

[jfd70163-bib-0029] Shin, D. J. , M. J. Kim , J. G. Min , and K. I. Kim . 2025. “Development of Viability‐Quantitative PCR With Propidium Monoazide for Assessment of White Spot Syndrome Virus Structural Integrity and Viability.” Aquaculture 602: 742317.

[jfd70163-bib-0030] Stone, D. M. , W. Ahne , K. L. Denham , et al. 2003. “Nucleotide Sequence Analysis of the Glycoprotein Gene of Putative Spring Viraemia of Carp Virus and Pike Fry Rhabdovirus Isolates Reveals Four Genogroups.” Diseases of Aquatic Organisms 53: 203–210. 12691191 10.3354/dao053203

[jfd70163-bib-0032] Taylor, S. C. , K. Nadeau , M. Abbasi , C. Lachance , M. Nguyen , and J. Fenrich . 2019. “The Ultimate qPCR Experiment: Producing Publication Quality, Reproducible Data the First Time.” Trends in Biotechnology 37: 761–774. 30654913 10.1016/j.tibtech.2018.12.002

[jfd70163-bib-0033] Thermo Fisher Scientific . n.d. “Real‐Time PCR: Understanding Ct. Applied Biosystems, Thermo Fisher Scientific.” https://www.thermofisher.com/.

[jfd70163-bib-0034] Thilakarathna, S. H. , T. Stokowski , and L. Chui . 2022. “An Improved Real‐Time Viability PCR Assay to Detect *Salmonella* in a Culture‐Independent Era.” International Journal of Molecular Sciences 23, no. 23: 14708. 36499040 10.3390/ijms232314708 PMC9738789

[jfd70163-bib-0049] Tholen, D. W. , A. Kallner , J. W. Kennedy , J. S. Krouwer , and K. Meier . 2004. “Evaluation of Precision Performance of Quantitative Measurement Methods; Approved Guideline—Second Edition.” Evaluation 24, no. 25.

[jfd70163-bib-0046] Untergasser, A. , I. Cutcutache , T. Koressaar , et al. 2012. “Primer3—New Capabilities and Interfaces.” Nucleic Acids Research 40, no. 15: e115. 10.1093/nar/gks596. 22730293 PMC3424584

[jfd70163-bib-0035] Van Holm, W. , J. Ghesquière , N. Boon , et al. 2021. “A Viability Quantitative PCR Dilemma: Are Longer Amplicons Better?” Applied and Environmental Microbiology 87, no. 5: e02653‐20. 33361365 10.1128/AEM.02653-20 PMC8090887

[jfd70163-bib-0036] Wang, J. , Y. Liu , L. Yu , et al. 2016. “Characterization of Grass Carp (*Ctenopharyngodon idellus*) Ovary Cell Line and Its Susceptibility to Spring Viremia of Carp Virus.” Progress in Fishery Science 37: 56–60.

[jfd70163-bib-0037] Ward, R. L. , D. R. Knowlton , and P. E. Winston . 1986. “Mechanism of Inactivation of Enteric Viruses in Fresh Water.” Applied and Environmental Microbiology 52: 450–459. 3021056 10.1128/aem.52.3.450-459.1986 PMC203555

[jfd70163-bib-0038] Waterhouse, A. M. , J. B. Procter , D. M. A. Martin , M. Clamp , and G. J. Barton . 2009. “Jalview Version 2: A Multiple Sequence Alignment Editor and Analysis Workbench.” Bioinformatics 25: 1189–1191. 19151095 10.1093/bioinformatics/btp033 PMC2672624

[jfd70163-bib-0039] World Organisation for Animal Health (WOAH) . 2019. “Chapter 1.1.2. Principles and Methods of Validation of Diagnostic Assays for Infectious Diseases.” OIE Terrestrial Manual.

[jfd70163-bib-0040] World Organisation for Animal Health (WOAH) . 2023. “Chapter 2.3.9. Infection With Spring Viraemia of Carp Virus (SVCV).” Aquatic Animal Health Code and Diagnostic Manual.

[jfd70163-bib-0041] Ye, J. , G. Coulouris , I. Zaretskaya , I. Cutcutache , S. Rozen , and T. L. Madden . 2012. “Primer‐BLAST: A Tool to Design Target‐Specific Primers for Polymerase Chain Reaction.” BMC Bioinformatics 13: 134. 22708584 10.1186/1471-2105-13-134 PMC3412702

[jfd70163-bib-0042] Yue, Z. , Y. Teng , C. Liang , et al. 2008. “Development of a Sensitive and Quantitative Assay for Spring Viremia of Carp Virus Based on Real‐Time RT‐PCR.” Journal of Virological Methods 152: 43–48. 18602422 10.1016/j.jviromet.2008.05.031

[jfd70163-bib-0043] Zeng, L. , J. Li , M. Lv , et al. 2023. “Environmental Stability and Transmissibility of Enveloped Viruses at Varied Animate and Inanimate Interfaces.” Environment & Health 1, no. 1: 1–20. 10.1021/envhealth.3c00005 PMC11504606 37552709

[jfd70163-bib-0044] Zhang, N. Z. , L. F. Zhang , Y. N. Jiang , T. Zhang , and C. Xia . 2009. “Molecular Analysis of Spring Viraemia of Carp Virus in China: A Fatal Aquatic Viral Disease That Might Spread in East Asia.” PLoS One 4, no. 7: e6337. 19623265 10.1371/journal.pone.0006337 PMC2710009

[jfd70163-bib-0045] Zhu, X. , K. Yang , J. Xie , et al. 2025. “An SDS‐NaOH‐Based Method to Isolate Genome of Recombinant Adeno‐Associated Virus Vectors for Physical Titer Measurement.” PLoS One 20, no. 4: e0315921. 40179116 10.1371/journal.pone.0315921 PMC11967980

