# Supplementary figures and images for: Development and Validation of a Viability RT‐qPCR Assay for Detecting Infectious Spring Viraemia of Carp Virus (SVCV)
Source: J Fish Dis. 2026 Mar 15;49(8):e70163. doi: 10.1111/jfd.70163 (PMC13331534; doi:10.1111/jfd.70163)

## Slide 1
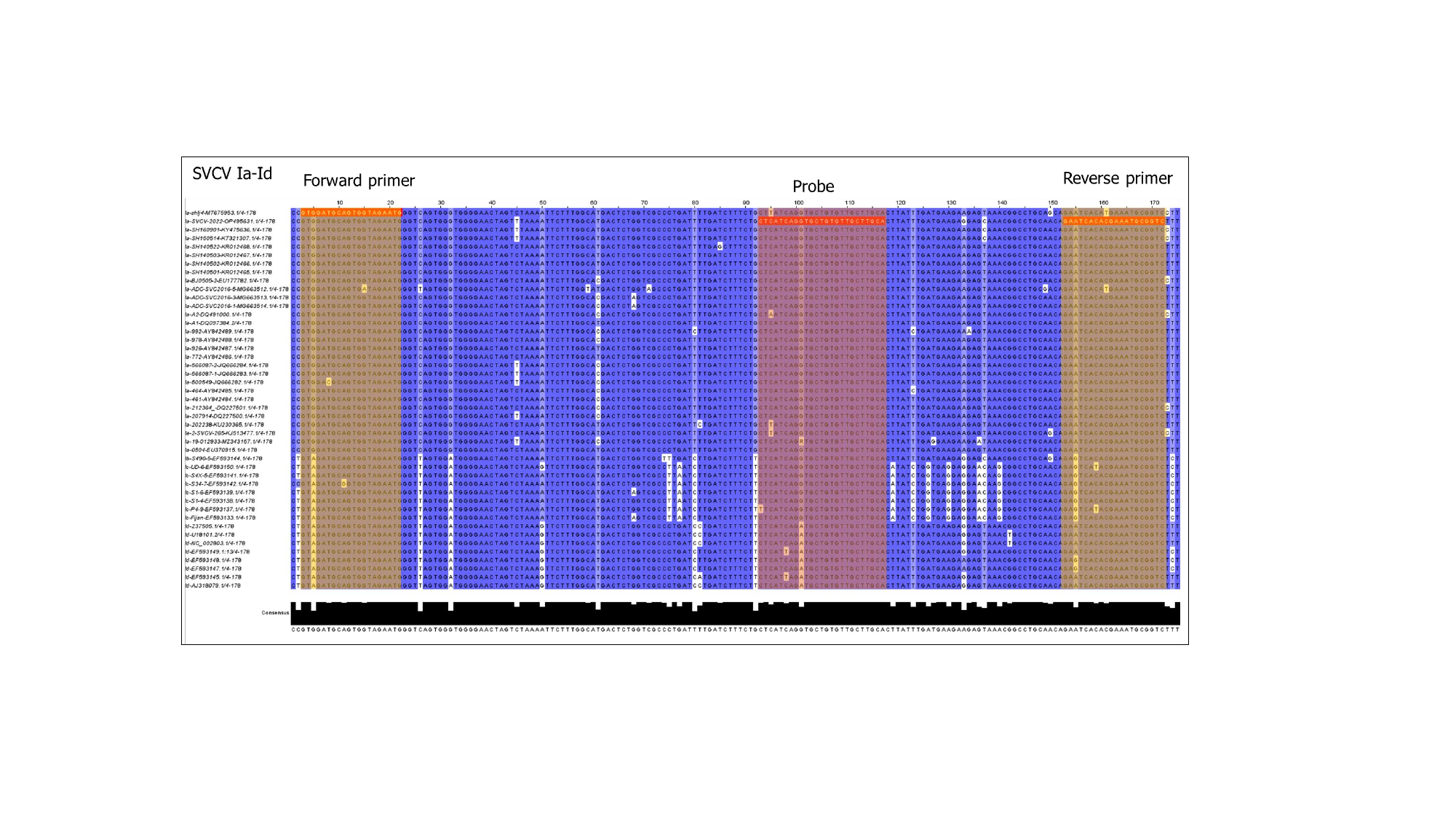

## Slide 2
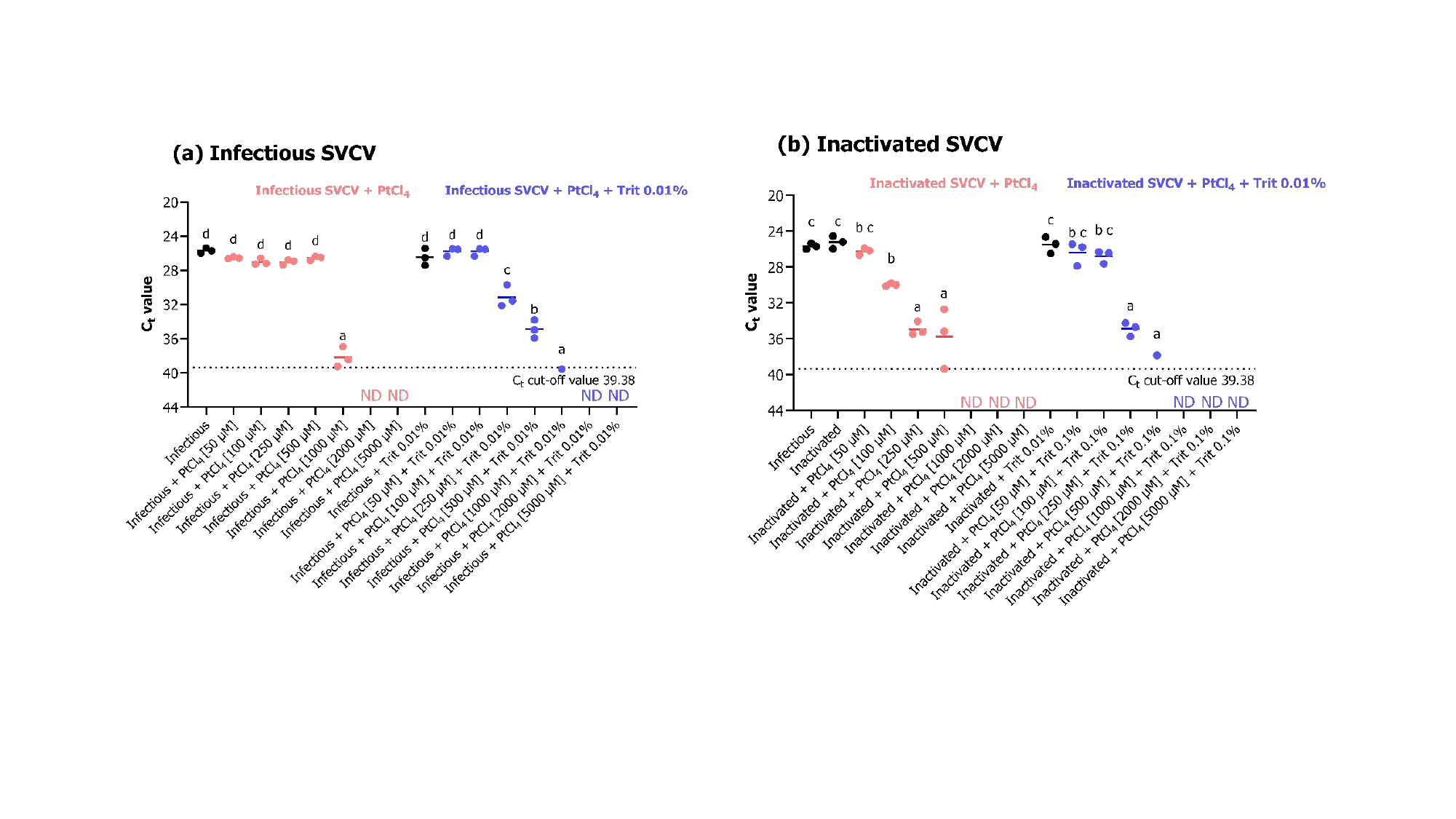

## Slide 3
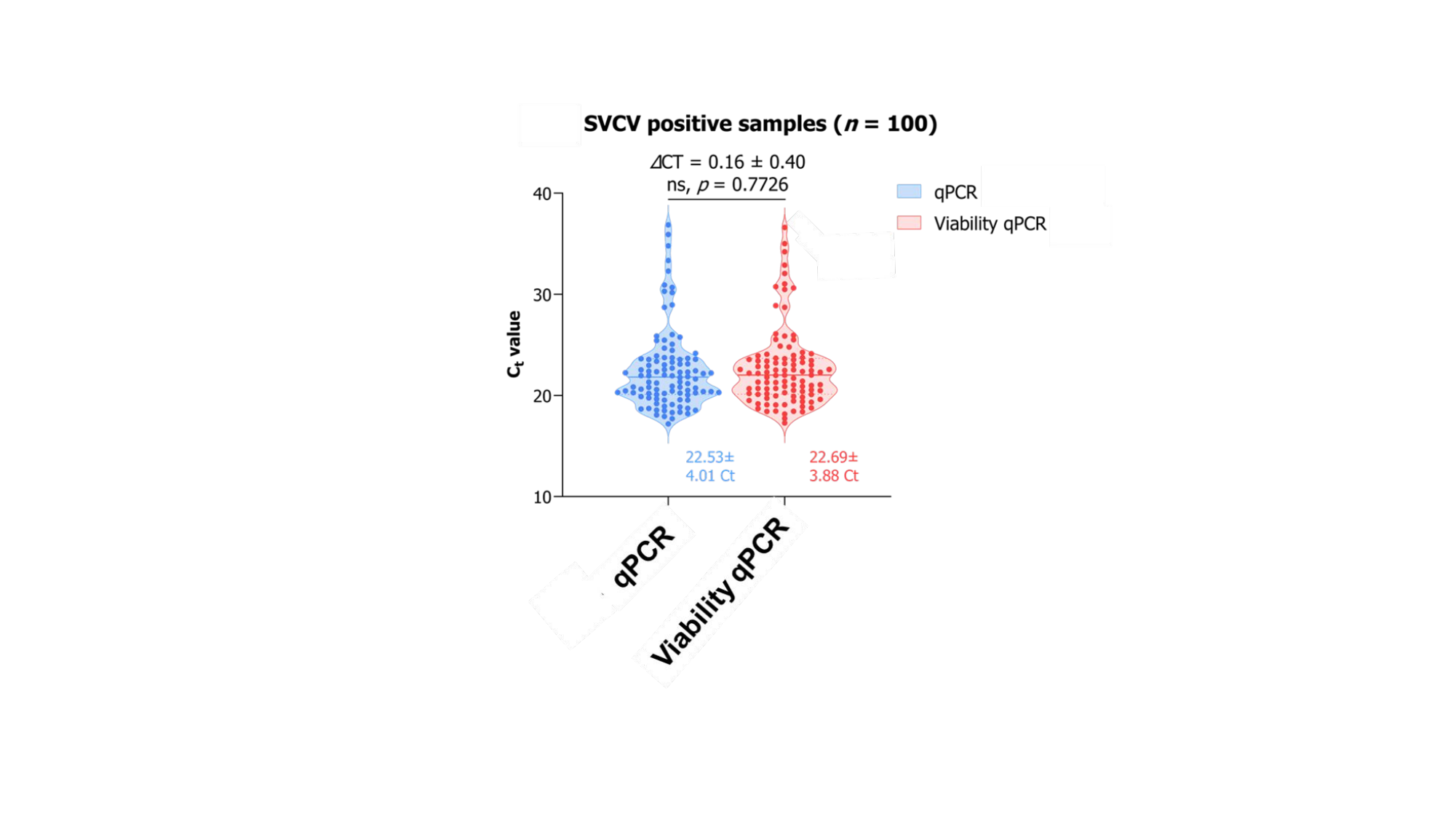

## Slide 4
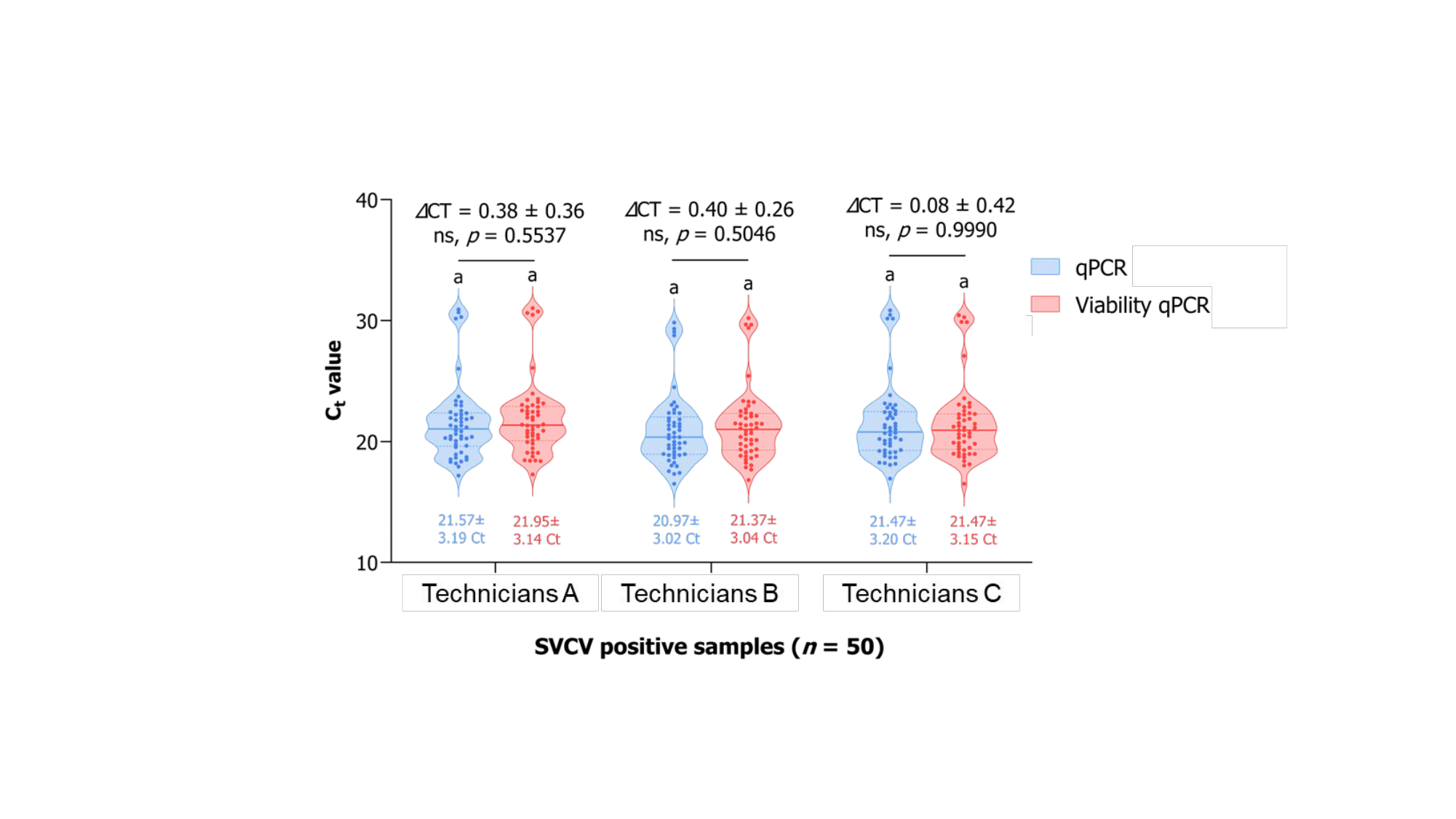

Supplement: Supplementary file 1 — Figures S1‐S4: jfd70163‐sup‐0001‐Supinfo.pptx. [file JFD-49-e70163-s001.pptx]
