# Supplementary material for: Development and Validation of a Viability RT‐qPCR Assay for Detecting Infectious Spring Viraemia of Carp Virus (SVCV)
Source: J Fish Dis. 2026 Mar 15;49(8):e70163. doi: 10.1111/jfd.70163 (PMC13331534; doi:10.1111/jfd.70163)
Supplement: Supplementary file 2 — Table S1: Performance evaluation of the spring viremia of carp virus (SVCV) quantitative PCR (qPCR) assay diagnostic assay at different annealing‐extension temperatures. Table S2: Limit of detection at 95% (LoD95%) of the spring viremia of carp virus (SVCV) quantitative PCR (qPCR) assay at different plasmid DNA (pDNA) concentrations. Table S3: Verification of the detection probability at 2× and 1× the 95% limit of detection (LoD95%) for the spring viremia of carp virus (SVCV) quantitative PCR (qPCR) assay. Table S4: Cross‐reactivity of the Spring viraemia of carp virus (SVCV) quantitative PCR (qPCR) assay with various aquatic pathogens. [file JFD-49-e70163-s002.docx]

**Table S1.** Performance evaluation of the spring viremia of carp virus (SVCV) quantitative PCR (qPCR) assay diagnostic assay at different annealing-extension temperatures.

| **Concentrations (copies/reaction)** | **56℃** | | **58℃** | | **60℃** | | | **62℃** | |
| --- | --- | --- | --- | --- | --- | --- | --- | --- | --- |
|  | **Mean C_t_**  **(SD)** | **Detection rate (%)** | **Mean C_t_**  **(SD)** | **Detection rate (%)** | **Mean C_t_**  **(SD)** | | **Detection rate (%)** | **Mean C_t_**  **(SD)** | **Detection rate (%)** |
| Neat | 18.02 (0.1) | 100 | 17.8 (0.19) | 100 | 17.27 (0.23) | 100 | | 17.8 (0.15) | 100 |
| 10^-1^ | 21.22 (0.1) | 100 | 20.98 (0.14) | 100 | 20.83 (0.04) | 100 | | 21 (0.47) | 100 |
| 10^-2^ | 24.93 (0.01) | 100 | 24.98 (0.12) | 100 | 24.75 (0.12) | 100 | | 25.63 (0.67) | 100 |
| 10^-3^ | 28.18 (0.1) | 100 | 28.15 (0.11) | 100 | 28.01 (0.09) | 100 | | 29.02 (0.84) | 100 |
| 10^-4^ | 31.39 (0.18) | 100 | 31.3 (0.03) | 100 | 30.76 (0.43) | 100 | | 31.73 (0.54) | 100 |
| 10^-5^ | 35.1 (0.27) | 100 | 34.89 (0.11) | 100 | 35.09 (1.11) | 100 | | 35.5 (1.57) | 100 |
| 10^-6^ | 36.71 | 33.3 | 37.49 (0.54) | 66.6 | 38.31 (0.51) | 66.6 | | 38.12 | 33.3 |
| Negative control (DEPC-DW) | Not detected | | Not detected | | Not detected | | | Not detected | |

**Table S2.** Limit of detection at 95% (LoD_95%_) of the spring viremia of carp virus (SVCV) quantitative PCR (qPCR) assay at different plasmid DNA (pDNA) concentrations.

| **Concentration**  **(copies/reaction)** | **Positive/total replicates tested**  **(%)** | **Mean C_t_**  **(SD)** | **C_t_ range**  **(min, max)** | **C_t_ CV**  **(%)** |
| --- | --- | --- | --- | --- |
| 1000 | 24/24 (100) | 31.72 (0.12) | 31.48 – 31.91 | 0.38 |
| 500 | 24/24 (100) | 32.64 (0.15) | 32.44 – 32.95 | 0.45 |
| 250 | 24/24 (100) | 33.79 (0.21) | 33.39 – 34.17 | 0.62 |
| 125 | 24/24 (100) | 34.86 (0.24) | 34.39 – 35.35 | 0.67 |
| 62.5 | 24/24 (100) | 35.70 (0.34) | 35.03 – 36.29 | 0.95 |
| 31.25 | 24/24 (100) | 36.86 (0.48) | 36.07 – 37.92 | 1.30 |
| 15.63 | 24/24 (100) | 37.66 (0.73) | 35.75 – 38.84 | 1.94 |
| 7.81 | 23/24 (95.83) | 38.60 (1.00) | 37.07 – 41.58 | 2.59 |
| 3.91 | 20/24 (83.33) | 39.80 (0.66) | 38.27 – 40.64 | 1.66 |
| 1.95 | 15/24 (62.50) | 40.42 (0.44) | 39.05 – 40.89 | 1.08 |
| Negative control (DEPC-D.W.) | Not detected | Not applicable | Not applicable | Not applicable |

**Table S3.** Verification of the detection probability at 2× and 1× the 95% limit of detection (LoD_95%_) for the spring viremia of carp virus (SVCV) quantitative PCR (qPCR) assay.

| **Concentration (copies/reaction)** | **Number of replicates tested (N)** | **Number of positive detected (N)** | **Positive rate (%)** | **Mean C_t_ (SD)** | **C_t_ CV (%)** |
| --- | --- | --- | --- | --- | --- |
| 14 copies (2× LoD_95%_) | 96 | 96 | 100 | 37.99 (0.76) | 1.99 |
| 7 copies (1× LoD_95%_) | 96 | 90 | 93.75 | 39.29 (0.97) | 2.47 |

**Table S4.** Cross-reactivity of the Spring viraemia of carp virus (SVCV) quantitative PCR (qPCR) assay with various aquatic pathogens.

|  | **Aquatic pathogens** | **SVCV signal** |
| --- | --- | --- |
| 1 | Spring viraemia of carp virus (SVCV; SVCV Ia type) | Positive |
| 2 | Red sea bream iridovirus (RSIV; RSIV type) | Negative |
| 3 | Infectious spleen and kidney necrosis Virus (ISKNV; ISKNV type) | Negative |
| 4 | Flounder iridovirus (FLIV; TRBIV type) | Negative |
| 5 | Lymphocystis disease virus (LCDV) | Negative |
| 6 | Viral nervous necrosis virus (VNNV; BFNNV type) | Negative |
| 7 | Viral hemorrhagic septicemia virus (VHSV; type IVa) | Negative |
| 8 | *Vibrio harveyi* (FP8370) | Negative |
| 9 | *V. ichthyoenteri* (FP8487) | Negative |
| 10 | *V. ordalii* | Negative |
| 11 | *V. campbellii* | Negative |
| 12 | *V. alginolyticus* | Negative |
| 13 | *V. anguillarum* (TR14001NF) | Negative |
| 14 | *Streptococcus iniae* (FP5228) | Negative |
| 15 | *S. parauberis* (FP3287) | Negative |
| 16 | *Photobacerium damselae* (FP4101) | Negative |
| 17 | *Lactococcus garvieae* (FP5245) | Negative |
| 18 | *Edwardsiella tarda* (FP5060) | Negative |
| 19 | *Escherichia coli* (JM109) | Negative |
| 20 | DEPC-DW | Negative |
